# A Qualitative Systematic Review of Barriers and Facilitators to Hepatitis B and C Programmes in Prisons

**DOI:** 10.1111/jvh.14049

**Published:** 2024-12-28

**Authors:** Heidi Emery, Catrin Evans, Kathryn Jack, Elisa Martello, Princella Seripenah, Fatima Aiyelabegan, Surakshya Dhungana, Titus Joseph, Dirontsho Koboto, Joanne R. Morling, James Stewart‐Evans, Emma Wilson, Jo Leonardi‐Bee

**Affiliations:** ^1^ Centre for Public Health and Epidemiology, School of Medicine University of Nottingham Nottingham UK; ^2^ Nottingham Centre for Evidence Based Healthcare, Faculty of Medicine and Health Sciences University of Nottingham Nottingham UK; ^3^ Nottingham University Hospitals NHS Trust Nottingham UK; ^4^ NIHR Nottingham Biomedical Research Centre Nottingham University Hospitals NHS Trust, and the University of Nottingham Nottingham UK

**Keywords:** barriers, facilitators, hepatitis, linkage to care, prison, systematic review

## Abstract

The prevalence of viral hepatitis among people in prisons is higher than in the general population. Screening, treatment and vaccination programmes exist within prisons to reduce the incidence of hepatitis, although lower uptake has often been reported compared to similar programmes outside of prisons. We conducted a systematic review of qualitative evidence to explore the barriers and facilitators to hepatitis B and C reduction programmes in prisons from the perspectives of people in prison, custodial staff and prison healthcare staff. Comprehensive searches of five databases (to November 2023) yielded 28 studies for review inclusion. Four synthesised findings were identified: (i) accurate, up‐to‐date knowledge of viral hepatitis disease and treatment among people in prison and staff is a facilitator to programme uptake, particularly when imparted by a trusted source; (ii) personal subjective and relative views have a bearing on participation with the programme; (iii) social interactions and relationships both within the community of people in prison and between them and staff groups influence participation in the programmes; and (iv) the organisational structure of the prison and healthcare services within it affect programme participation. Based on these findings, we make recommendations for the adaptation of viral hepatitis programmes to individual custodial settings thereby improving equitable programme access and hepatitis B and C reduction in this complex environment.

**Abbreviations**
BBV: blood‐borne virus
CS: custodial staff
DAAs: direct‐acting antivirals
HBV: hepatitis B Virus
HCV: hepatitis C Virus
HS: healthcare staff
JBI: Joanna Briggs Institute
PIP: people in prison

## 1 Introduction

The large global health burden of viral hepatitis‐related morbidity and mortality (mainly blood‐borne viral types B and C) has underpinned the World Health Organisation's (WHO) goal, endorsed by member states, to reduce new infections by 90% and deaths by 65% between 2016 and 2030 through prevention strategies, testing and treating initiatives and increased service coverage [1, 2].

People in prison (PIP) are disproportionately affected by blood‐borne hepatitis. Hepatitis C virus (HCV) infection prevalence worldwide in prisons is estimated to range from 10% to 30% [3] compared to approximately 2% in the general population [4] and hepatitis B virus (HBV) prevalence is 5% for PIP and 4% for the general population [5, 6]. HCV and HBV are transmitted through exposure to blood products (including injected drug use), sexual contact and vertically (from mother to child) [7], thus there is an increased vulnerability among people who inject drugs and sex workers. These populations often face barriers to healthcare in the community and since the behaviours are criminalised, they comprise a higher proportion of prison populations than in the community leading to the higher prison hepatitis prevalence [8]. Once incarcerated, the use of nonsterile injecting equipment, other bodily fluid contact and inequitable access to harm reduction services perpetuates transmission [9].

To meaningfully reduce population prevalence, the WHO recommends PIP should receive targeted hepatitis reduction programmes. These comprise (i) equitable access by the removal of structural barriers to healthcare, (ii) the availability of proven hepatitis prevention interventions (needle and syringe programmes, opiate substitution and HBV vaccination), testing and treatment and (iii) access to broader health interventions for the co‐morbidities that frequently occur in this population and impact programme uptake [10]. However, when harm reduction, screening and treatment programmes are available within prisons, lower uptake has often been reported compared to those in the wider population [11, 12, 13, 14]. Qualitative research can provide insights into why PIP may, or may not, engage with these programmes. Studies have been undertaken to explore this phenomenon, but a systematic synthesis of these has not been performed. Prisons are complex environments, previously described as functioning like communities [15]; hence, exploration from the differing perspectives of the principal stakeholders within the setting will deepen understanding of the phenomenon. Therefore, we conducted a systematic review of qualitative evidence to evaluate the barriers and facilitators to HBV and HCV reduction programmes in prisons from the perspectives of PIP, custodial staff and prison healthcare staff, with the objective of enhancing their implementation.

## 2 Methods

This qualitative systematic review was conducted using the JBI meta‐aggregative methodological approach [16] and reported according to the Enhancing Transparency in Reporting Synthesis of Qualitative Research (ENTREQ) guidance [17]. The protocol was registered with PROSPERO (CRD42023481096) [18].

Studies were included if they met the following criteria:

Participants: PIP, prison healthcare staff and custodial staff of any age or gender. We excluded studies where the views described were those of stakeholders not working directly at the prison facility, for example, policymakers.

Phenomena of interest: Experiences of the barriers and facilitators to HBV and HCV reduction programmes in prisons as described by PIP, custodial staff and prison healthcare staff.

Context: Any country and any type of correctional custodial facility.

Study design: Any design containing qualitative data obtained from, but not limited to, interviews, focus groups and unstructured questionnaires were considered. Qualitative data within quantitative surveys was considered where open questions relating to the phenomena of interest had been included.

### 2.1 Search Strategy

An initial limited search of MEDLINE (via Ovid) was undertaken with analysis of text and index terms used to describe key articles. These were developed into a comprehensive search strategy with assistance from a research librarian which was used and adapted as necessary across the databases, see Appendix S1. The reference lists of all included articles were hand searched for additional studies.

The electronic databases Medline (Ovid), Embase (Ovid), CINAHL, APA PsycINFO (Ovid) and CINAHL (EBSCoHost) were searched in November 2023 for studies published from 2000 onwards to capture the range of experiences across changing treatment availability; interferon‐based treatments were available from 2000 and direct‐acting antiviral treatments (DAAs) from 2011 onwards [7]. Google Scholar was searched for grey literature using the same date range and adapted search strategy.

Studies in all languages were considered. Non‐English texts were translated using DeepL Translator software (www.DeepL.com/translator) with further professional translation if required.

### 2.2 Screening of Studies

Studies identified from the searches were uploaded into Covidence [19] and de‐duplicated. Two reviewers (HE and PS) independently screened all titles and abstracts, with discrepancies resolved through discussion with a third reviewer (JL‐B). Full texts of potentially eligible studies underwent the same process.

### 2.3 Data Extraction and Critical Appraisal

Extracted data related to study characteristics and study findings (with accompanying illustrations) were entered into a previously piloted spreadsheet (Appendix S2). Following a piloting of extracted data for five studies by two reviewers (HE and JL‐B), the remaining studies were extracted by one reviewer (HE). Study findings were verbatim extracts of the study author's analytical interpretation of the results of the data. Each finding was assigned a level of credibility (unequivocal, credible and not supported) dependent on the congruency between the data and the accompanying illustration [16]. Studies were critically appraised using the JBI Checklist for Qualitative Research [16]. Appraisal was initially conducted by two reviewers (HE and JL‐B) for five studies, with a high level of agreement being seen, therefore the remaining studies were critically appraised by one reviewer (HE).

### 2.4 Data Synthesis and Confidence in the Findings

Findings assigned as either unequivocal or credible were grouped into categories based on similarity in meaning and concept through discussion by four reviewers (HE, FA, EM and JL‐B). Findings rated as not supported were not synthesised [16]. Further joint discussions (HE, JL‐B and CE) refined the categories and aggregated them into synthesised findings which could be used for evidence‐based practice. Findings from each separate participant group were initially analysed separately. However, joint discussions (HE, JL‐B and CE) identified strong similarities in meaning across the different groups. Hence, the review findings are presented as an overall synthesis in which the findings from different groups are combined. A collaborative process was used to establish a level of confidence in each synthesised finding using the JBI ConQual approach [20]. The confidence ranking was derived from the dependability of the studies and the credibility of individual included findings (full details in Table 6).

## 3 Results

### 3.1 Study Inclusion and Quality Assessment

The database search returned 662 records, of which 256 records were duplicates. Title and abstract screening were performed in 407 studies, of which 45 were potentially relevant and underwent full‐text screening. Hand‐searching reference lists of these returned one further study. Twenty‐eight studies were eligible for inclusion in the review (Table 1). The remaining 17 studies were excluded based on ineligibility of study design (8 studies), context (1 study), phenomena of interest (7 studies) or participants (1 study) (Appendix S3).

**TABLE 1:** Characteristics of included studies with critical appraisal.

Lead Author (Reference)	Year	Title	Country	Setting	Methodology	Method	Participants	Data collection period	Phenomena of interest	Intervention studied	JBI Checklist Score
Akiyama [21]	2020	Knowledge, attitudes and acceptability of direct‐acting antiviral hepatitis C treatment among people incarcerated in jail: A qualitative study	USA	New York City Jails (awaiting or undergoing trial)	Iterative thematic analysis	Semi‐structured in‐person interviews	36 inmates 21 males 15 females All HCV positive	Mar— April 2015	Participants’ knowledge of HCV and HCV treatment as well as the acceptability of HCV treatment among jail detainees	DAA	10
Byrne [22]	2023	Mixed‐methods evaluation of point‐of‐care hepatitis C virus RNA testing in a Scottish prison	Scotland	1 x male maximum security prison, Perth	Mixed Methods. Deductive thematic analysis. Themes allocated to predetermined domains	Semi‐structured in‐person interviews. 2 x Focus groups for nursing staff.	6 NHS healthcare staff members	Dec 2018 —Mar 2021	Barriers and facilitators to implementation of point‐of‐care RNA testing	Point‐of‐care HCV RNA testing (fingerstick) compared to conventional phlebotomy and dried blood spot	7
Crowley [23]	2018	Barriers and facilitators to hepatitis C (HCV) screening and treatment—a description of prisoners' perspective	Ireland	2 x Medium security prisons one male, one female, Dublin	Grounded theory: collection and analysis with thematic coding	4 x Focus groups Guideline of open‐ended questions	46 prisoners male:female ratio unknown	2017	Barriers and facilitators to HCV screening and treatment	Screening (dried blood spot testing and liver scan) drug therapy (DAA)	8
Crowley [24]	2018	Hepatitis C virus screening and treatment in Irish prisons from a governor and prison officer perspective—a qualitative exploration.	Ireland	Governor component = 13 prisons Prison officer component = 2 prisons: one male, one female	Grounded theory: collection and analysis with thematic coding	5 x Focus groups in total 3 x prison governor 2 x prison officer Guideline of open‐ended questions	13 Governors 55 prison officers	3 months in 2017	To explore prison governors' and officers' views on barriers and enablers to HCV screening and treatment.	HCV screening. Type not specified	8
Crowley [25]	2019	Hepatitis C virus screening and treatment in Irish prisons from nurse manager's perspectives—a qualitative exploration.	Ireland	12 closed prisons	Grounded theory: Collection and analysis with thematic coding	1 x Focus group. Guideline of open‐ended questions	12 nurse managers	Not reported	To explore prison nurse manager's perspectives on hepatitis C virus screening and treatment in Irish prisons.	HCV screening (mainly phlebotomy based)	9
Crowley [26]	2019	Competing priorities and second chances—A qualitative exploration of prisoner's journeys through the hepatitis C continuum of care.	Ireland	1 x Medium security prison, Dublin	Grounded theory: collection and analysis with thematic coding	One‐to‐one semi‐structured in‐person interviews	25 male prisoners All with chronic HCV infection	July—Nov 2018	Prisoner's narratives of their HCV journey.	HCV screening and care. Interferon based and DAA.	9
Dyer [27]	2009	Hepatitis C education and support in Australian prisons: preliminary findings of a nationwide survey.	Australia	Prisons from all states of Australia. Number not stated.	Action research framework.	Semi‐structured telephone interviews	23 healthcare personnel	Not reported	To explore the efficiency of hepatitis C education and support services available in custodial settings from the perspective of health educators and policy makers.	HCV education and harm reduction measures	8
Heinemann [28]	2001	Prevention of blood‐borne virus infections among drug users in an open prison by syringe vending machines.	Germany	1 x open prison Hamburg‐Vierland	Hermeneutic analysis, hypotheses generated	Guided in‐person interviews	9 x prison staff 22 x male inmates	1996	The feasibility and acceptability of a pilot project for the automated dispensing of disposable syringes to inmates who use drugs.	Sterile syringe dispensing	5
Jack [29]	2017	Prison officers' views about hepatitis C testing and treatment: a qualitative enquiry.	England	1 x Category B male prison in the East Midlands area	Phenomenology, with thematic analysis	Semi‐structured in‐person interviews	10 x prison custody officers 8 x men 2 x women	July—Aug 2014	To explore the views of prison custody officers about prisoners being tested and treated for HCV.	HCV screening and treatment. Type not specified.	10
Jack [30]	2020	How do people in prison feel about opt‐out hepatitis C virus testing?	England	1 x low security prison, East Midlands	Thematic network approach	Semi‐structured in‐person interviews.	45 male prisoners	Not reported	The perceptions and experiences of people in prison to the implementation of an opt‐out blood‐borne virus test intervention.	Screening by dried spot blood test	10
Jacob [31]	2000	The transfer of harm reduction strategies into prisons: Needle exchange programmes in two German prisons.	Germany	2 x prisons Lower Saxony 1 x male 1 x female	Not reported	Group interview.	Prisoners, healthcare and custodial staff Number not reported. Mixed male and female	1996	The feasibility, usefulness and efficacy of harm reduction strategies for inmates who use drugs.	The supply of sterile syringes to prisoners.	5
Kamat [32]	2023	Access to hepatitis C treatment during and after incarceration in New Jersey, United States: A qualitative study	USA	Re‐entry facility, New Jersey	Iterative thematic analysis	Semi‐structured in‐person interviews	27 prisoners: 22 x male, 5 x female All HCV positive	July 2020–July 2021	Barriers and facilitators to HCV treatment uptake during and after incarceration	DAA	10
Khaw [33]	2007	‘I just keep thinking I haven't got it because I'm not yellow’: a qualitative study of the factors that influence uptake of hepatitis C testing by prisoners.	England	3 x prisons North East	Themes developed from constant comparative analysis	Semi‐structured in‐person interviews	30 prisoners 25 x male 5 x female All previous IDUs	Not reported	To identify the factors that influence the uptake of testing for HCV infection by prisoners.	HCV screening via venepuncture (pre‐DAA)	8
Lafferty [34]	2017	Social capital strategies to enhance hepatitis C treatment awareness and uptake among men in prison	Australia	3 x correctional centres New South Wales	Latent‐level thematic analysis	In‐person interviews Appreciative enquiry across common dimensions of social capital	28 male inmates All HCV RNA positive or receiving HCV treatment	Nov 2014 to March 2015	To explore features of social capital and their relation to HCV education and treatment among inmates living with HCV.	Interferon + ribavirin (pre‐DAA)	8
Lafferty [35] Also Lafferty [36]^a^ Also Rance [37]^a^	2018 2019 2020	Understanding facilitators and barriers of direct‐acting antiviral therapy for hepatitis C virus infection in prison ‘Fighting a losing battle’: Prisoners' perspectives of treatment as prevention for hepatitis C with inadequate primary prevention measures. ‘Behind closed doors, no one sees, no one knows’: hepatitis c, stigma and treatment as prevention in prison.	Australia	4 x prisons: 3 x male 1 x female New South Wales	Latent thematic analysis	Semi‐structured in‐person interviews	32 prisoners 24 x male 8 x female All IDU (12 previously received interferon treatment)	Not reported.	To understand the perceived barriers and facilitators for the delivery of HCV treatment in prison in contrast to community‐based treatment.	DAA	8
Lafferty [38] Also: Lafferty [39]^a^	2020 2021	Perceptions and concerns of hepatitis C reinfection following prison‐wide treatment scale‐up: Counter‐public health amid hepatitis C treatment as prevention efforts in the prison setting. The role of social capital in facilitating hepatitis C treatment scale‐up within a treatment‐as‐prevention trial in the male prison setting.	Australia	4 x prisons, New South Wales	Thematic analysis	Semi‐structured in‐person interviews	23 male prisoners, all completed HCV treatment	Not reported	The perceptions of HCV reinfection following ‘cure’ among people in prison.	Post DAA treatment	9
Lafferty [40] Also: Lafferty [41]^a^	2022 2021	‘You need a designated officer’—Recommendations from correctional and justice health personnel for scaling up hepatitis C treatment as prevention in the prison setting. Hepatitis C treatment as prevention in the prison setting: Assessments of acceptability of treatment scale‐up efforts by prison correctional and health personnel.	Australia	4 x prisons: 3 x male 1 x female New South Wales	Thematic analysis to a predefined framework	Semi‐structured in‐person interviews	24 x correctional personnel 17 x health personnel	March–April 2018	To examine recommendations by correctional and justice health personnel for HCV treatment‐as‐prevention scale up in the prison setting.	DAA	9.5
Lafferty [42] Also: Lafferty [43]^a^	2023 2022	Reducing barriers to the hepatitis C care cascade in prison via point‐of‐care RNA testing: A qualitative exploration of men in prison using an integrated framework. That was quick, simple and easy: Patient perceptions of acceptability of point‐of‐care hepatitis C RNA testing at a reception prison.	Australia	1 x reception prison, New South Wales	Thematic analysis to a predefined framework	Semi‐structured in‐person interviews	24 x male prisoners, all undergone HCV testing	Nov 2020—April 2021	To understand the role of point‐of‐care HCV RNA testing at intake in reducing barriers to the HCV care cascade within the male prison setting.	Point‐of‐care HCV RNA screening	8
Long [44]	2004	Prisoners' views of injecting drug use and harm reduction in Irish prisons	Ireland	2 x prisons Dublin area	Grounded theory thematic analysis	Semi‐structured in‐person interviews	31 x male prisoners 16 x IDU 15 x non‐IDU	2000 5 weeks	To examine prisoners' views of drug‐injecting practices and harm reduction interventions in Dublin prisons.	Standard harm reduction measures available.	9.5
Ly [45]	2018	Perspectives on integrated HIV and hepatitis C virus testing among persons entering a Northern Carolina Jail: A pilot study	USA	1 x jail Northern Carolina	Thematic analysis	Semi‐structured in‐person interviews	30 inmates 27 x male 3 x female	Sept— Nov 2015	To explore factors influencing HIV and HCV testing decisions and individual's preferences and concerns regarding opt‐in vs. opt‐out testing at the time of jail entry.	HCV screening. Type not specified	7.5
Miller [46]	2021	People in prison who inject drugs: Who is trusted when it comes to information about hepatitis C?	Australia	Unclear number of prisons, New South Wales	Content analysis stated but thematic analysis described.	Semi‐structured in‐person interviews	30 x prisoners 20 x males 10 x females	2013–2014	Interpersonal trust within the relationships between people in prison and other individuals from whom they access information regarding HCV.	Pre‐DAA	8
Mina [47]	2016	Hepatitis C in Australian prisons: A national needs assessment.	Australia	Prisons from all states of Australia	Mixed Methods. Findings matched themes from a previous workshop.	Semi‐structured in‐person interviews	55 Stakeholders including 32 service delivery healthcare personnel	Jan—Jun 2014	To examine barriers and opportunities for development of infrastructure for enhanced services.	Interferon + ribavirin (pre DAA)	6.5
Munoz‐ Plaza [48]	2005	Hepatitis C service delivery in prisons: Peer education from the ‘Guys in Blue’.	USA	1 x prison California	Thematic analysis	Prisoners: 1 x focus group (*n* = 6) and individual semi‐structured in‐person interviews (*n* = 5) Staff: in‐person interviews	11 x male prisoners in a drug treatment programme	2003	To describe the HCV services offered at a corrections‐based drug treatment programme; client and staff perceptions of the advantages, benefits and barriers to delivering existing services and their recommendations for enhancing services.	HCV education	8
Neuhaus [49]	2018	Telementoring for hepatitis C treatment in correctional facilities.	Australia	5 x prisons Queensland	Content analysis stated but thematic analysis described.	Semi‐structured interviews. In‐person or telephone.	16 x health staff	2017	The clinical effectiveness and other impacts from the perspective of service staff involved in the HCV telementoring service.	A telementoring service to upskill doctors and nurse practitioners. Post‐DAA	7.5
Rehman [50]	2004	Harm reduction and women in the Canadian National Prison System: Policy or practice?	Canada	9 x prisons	Constant comparison to search for emergent themes.	Semi‐structured in‐person interviews	156 x female prisoners	2001–2002	The perceptions and lived experiences of a sample of nationally incarcerated women in Canada regarding their perceptions and experiences in accessing HIV and Hepatitis C prevention, care, treatment and support.	Harm reduction measures	8
Thornton [51]	2018	The New Mexico Peer Education Project: Filling a critical gap in HCV prison education	USA	7 x prisons New Mexico	Inductive thematic analysis	7 x Focus groups and 19 x semi‐structured in‐person interviews	76 x prisoners 57 in focus groups (male) 19 interviewed (3 x female)	2012–2016	To describe an HCV peer education project and its impact on peer educators and their students.	Peer education project	8.5
Wurcel [52]	2021	‘I'm not gonna be able to do anything about it, then what's the point?’: A broad group of stakeholders identify barriers and facilitators to HCV testing in a Massachusetts jail.	USA	1 x jail, Massachusetts	Grounded theory, thematic analysis.	Semi‐structured in‐person interviews	21 x male prisoners and 9 x clinicians	Nov 2018—April 2019	To better understand barriers and opportunities for HCV testing.	DAA	10
Yap [53]	2014	A descriptive model of patient readiness, motivators and hepatitis C treatment uptake among Australian prisoners.	Australia	16 x Prisons in 3 states.	Grounded theory, thematic analysis. Prisoner's data triangulated with perspectives from health service providers.	Semi‐structured, in‐person interviews	116 prisoners who had refused, deferred, delayed or discontinued HCV treatment, 89 × male, 27 × female. 29 Health professionals.	2010–2013	To explore factors affecting treatment uptake inside prisons.	Pre‐DAA	8

Abbreviations: DAA = Direct‐acting antiviral, HCV = hepatitis C virus, HIV = human immunodeficiency virus, IDU=injecting drug user, RNA = ribonucleic acid.

^a^ Findings from the same interview data were reported across multiple papers and treated as one study for this review.

Most studies (*n* = 22) were assessed as being of moderate to high quality, scoring between 8 and 10 [21, 23, 24, 25, 26, 27, 29, 30, 32, 33, 34, 35, 38, 40, 42, 44, 46, 48, 50, 51, 52, 53], with six studies of lower quality [22, 28, 31, 45, 47, 49] (Table 1; Appendix S4). A key weakness across many studies was limited reporting of reflexivity.

### 3.2 Characteristics of Included Studies

All studies were conducted in high‐income countries (Table 1). Two used mixed methodology with the others solely qualitative. The majority (*n* = 20) employed a form of grounded theory or thematic analysis with five studies using framework analysis. Twenty studies reported interviews with PIP (10 male and female [21, 23, 31, 32, 33, 35, 45, 46, 51, 53], nine male only [26, 28, 30, 34, 38, 42, 44, 48, 52] and one female only [50]), nine reported interviews with prison healthcare staff [22, 25, 27, 31, 40, 47, 49, 52, 53] and five reported interviews with custodial staff [24, 28, 29, 31, 40]. Six studies reported interviews with more than one category [28, 31, 40, 47, 52, 53]. Study data were collected between 1996 and 2020. The sample sizes of the studies ranged from 6 to 156.

Four studies focused on generalised viral hepatitis harm reduction measures [28, 31, 44, 50]. Twenty‐four studies focused on screening and treatment for HCV, nine of these when interferon‐based treatments were available [27, 33, 34, 45, 46, 47, 48, 51, 53] and 15 when direct‐acting antiviral treatments were being used in the setting [21, 22, 23, 24, 25, 26, 29, 30, 32, 35, 38, 40, 42, 49, 52]. No studies were specific to HBV.

### 3.3 Review Findings

The 28 studies in this review included 104 findings from the experiences of PIP, 36 findings from the experiences of prison healthcare staff and 24 findings from the experiences of custodial staff (Appendix S5). The findings were aggregated into 20 categories which were further interpreted into four synthesised findings. The categories underpinning the synthesised findings are presented in Tables 2, 3, 4, 5 and Appendix S5.

**TABLE 2:** Synthesised Finding 1.

Synthesised Finding 1	Categories	Example illustrations
Accurate, up‐to‐date knowledge of hepatitis disease and treatment among people in prison and staff is a facilitator to uptake in hepatitis programmes, particularly when imparted by a trusted source.	1: Poor awareness of hepatitis disease, testing and treatment availability among PIP limited intervention uptake	‘One said he wouldn't be interested because he needed to learn more about it. They need to tell me “it's for this and that”. They need to tell me about the complications’ (PIP) [52]. p4 PIP may only learn about treatment availability months or years after arriving in prison (PIP) [53]. p4
	2: Low intervention uptake was due to poor staff knowledge or skills.	‘[There is] very low interest in hepatitis, probably because they [staff] don't know much about it and it doesn't have the hype of HIV’ (PIP) [48]. p362 ‘There is poor education and awareness, and a lot of inexperience regarding managing complex health issues (such as hepatitis C)’. (HS) [47]. p6 ‘There's a problem of skill mix. Lots of nurses don't take bloods. If you have nobody trained up that's a problem. Some nurses are afraid to take bloods’. (HS) [25]. p3
	3: People in prison can fear hepatitis due to incorrect knowledge.	‘They might pass it on to them, it's like AIDS, when AIDS first came out, people thought they can't share a cup with someone, can't touch them, you can't be near them … it's a threat to me’. (PIP) [30]. p4
	4: People in prison can be fearful of starting treatment due to poor or out‐of‐date knowledge.	‘They used to get the injection but it made her sick or something. It did. She said she was very sick. Some people say “I'd rather die than do the treatment”’. (PIP) [23]. p3
	5: People in prison were more likely to believe information when from a credible source.	‘A lot of staff, they know what we do in here, but nobody is going to listen to them, but if its another guy in blue sits down with him, he'll go, “Oh, now that hits”’. (PIP) [48]. p355 ‘Who do you trust about hepatitis C information? Probably the clinic. Pamphlets, to a lesser extent. Like more on the public health nurse, yeah, I'd probably trust the most’. (PIP) [46]. p249

**Synthesised Finding 1: Accurate, up‐to‐date knowledge of HBV and HCV disease and treatment among PIP and staff is a facilitator to uptake in viral hepatitis programmes, particularly when imparted by a trusted source**.

This finding was synthesised from five categories, which included 17 findings from interviews with PIP, seven from healthcare staff (HS) and six from custodial staff (CS) (Table 2; Appendix S5). The synthesised finding highlights the importance of viral hepatitis disease and treatment knowledge, in which accurate, up‐to‐date knowledge of HBV and HCV disease and treatment among PIP and staff is a facilitator to intervention uptake, particularly when imparted by a trusted source.

Poor awareness of viral hepatitis disease, its prevalence and long‐term health effects along with testing and treatment availability limited intervention uptake among PIP [52, 53]. When PIP's hepatitis knowledge was inaccurate, particularly about hepatitis acquisition, it led to fear and prejudiced behaviour from enacted stigma which discouraged testing [30]. Poor or out‐of‐date knowledge of treatments led to fear of treatment initiation since previous interferon‐based regimes were longer, more invasive and with greater side effects than modern DAA programmes [23]. The method of imparting knowledge was also important. PIP were more likely to believe information when from a credible source, which may be healthcare or trusted peers but rarely custodial staff [46, 48].

Poor staff knowledge and skills were also barriers to programme participation. When staff members lacked up‐to‐date, accurate knowledge appropriate interventions were not promoted [48]. Viral hepatitis was regarded as a complex health issue requiring specialist skills such as blood taking which the available workforce may lack [25, 47].

**Synthesised Finding 2: Personal subjective and relative views have a bearing on viral hepatitis programme participation**.

The finding was synthesised from six categories comprising 30 findings from interviews with PIP, three from healthcare staff and two from custodial staff (Table 3; Appendix S5). This synthesised finding incorporates personal subjective and relative views which have a bearing on programme participation. These encompass the person in prison's perceptions of their own identity and self‐worth, health beliefs, ability to act on intention, previous experiences, competing priorities and enactment of choice. For staff, this incorporates personal views of health risks. Each person's own value system is imported into the prison environment and reflected in their behaviours.

**TABLE 3:** Synthesised Finding 2.

Synthesised Finding 2	Categories	Example illustrations
Personal subjective and relative views have a bearing on hepatitis programme participation.	1: Felt stigma is a barrier.	‘There's a stigma to someone on gear, but there is a bigger stigma to someone on gear that used needles … that's the way, and it's like, people who use cocaine look down on someone who uses heroin. People who use heroin look down on someone that's injecting heroin. Then people who are injecting heroin with hepatitis c look down on someone who has HIV and it's just mad’. (PIP) [26]. p7 ‘The combined shame associated with being in prison, a drug user and hepatitis infection can also lead a person to believe they are undeserving of treatment’. (PIP) [52]. p6
	2: A person's self‐perception of their ability to take part in the programme influences their decision to participate.	‘The ease of fingerstick testing was viewed as widely achievable. It is a good thing because there are a lot of drug users that do have trouble finding veins that would be in the same boat as me, that would not go and get a blood test, just because of how hard it is to find a vein’. (PIP) [42]. p4 ‘Yeah, it was easy, because you could tell me straightaway you know what I mean, whether … and I wasn't sitting around doing head miles’. (PIP) [42]. p3
	3: Self‐perception of health and the associated relevance of hepatitis influences a person's decision to participate in an intervention.	‘[How come you're not as interested?] Probably because I feel healthy. I don't know, [… but you said you wanted to do a liver function test.] Yeah, I just wanted to see if it was bad. Because if it is getting real bad then I'll see somebody about it, you know. Maybe get into treatment or whatever. But it isn't really giving me any problems’ (PIP) [53]. p6 ‘Yeah who do the [HCV] treatment, like I guess what they say is, “we'll do the treatment, but there's no use really doing the treatment, because we're just going to continue to shoot up’” (PIP) [38]. p4 ‘I worry about myself … with the increased violence actually …, we are exposed more and more to open wounds and stuff’. (CS) [24]. p.4. ‘Look [hep C treatment as prevention is] good for the jail. Like I think if you can keep hep C out, you're going to stop worries about needlestick injuries here’. (CS) [40]. p3
	4: Engagement with an intervention is dependent on the personal experience.	‘It's like, that was taken away from me. And it's like, there's another chance for it now if you want it, and I've never wanted to get clean as much as I do now, you know what I mean’. (PIP) [26]. p9 ‘The only concern I have is what the treatment entails—if I'm going to be sick from it; if I'm going to be weak from it. Those are things I need to worry about because I'm in a environment that I can't really depend on right now. I gotta have an awareness that's different from the street.’ (PIP) [21] p6
	5: Competing priorities are facilitators and barriers.	‘… a lot of them don't want to start treatment especially while they're on remand because of the stress of court … They want to be able to present well when they go to court’. (PIP) [53]. p4 ‘If you know there's a cure…You know, I have five daughters and now I got three grankids. So now I hope—I want to stay alive a little longer. Got some hope…I got grandkids I said…I'd love to be with them’. (PIP) [21]. p8
	6: Choice in healthcare can be an expression of freedom.	‘It's (healthcare) the only freedom that we've got. Something like that (BBV test) if it's an option, and you're trying to force it onto somebody, some of them will just go against it just for the sake of going against it, anti‐establishment’. (PIP) [30]. p5

The association of hepatitis with injecting drug use may lead a person to fear they will be thought less of by others if they have acquired the illness, which in turn leads to internalised feelings of fear and shame. Categorised as felt stigma, this acts as a barrier to testing uptake [26, 52].

Self‐perception of health and personal relevance of hepatitis influences the initiation of programme participation. For the PIP, this encompasses self‐perception of their current health status, possibly not starting treatment if they considered their HCV was not severe enough or considering treatment pointless if they were continuing drug use [53]. HCV infection could be viewed as benign or lacking in relevance to the individual due to a lack of symptoms [26, 32, 45]. Other PIP did not participate in interventions due to a fatalistic view of treatment not being worthwhile and a high probability of reinfection [38]. Custodial staff were more likely to promote and engage with programmes when they perceived their personal health was at risk by exposure to blood‐borne viruses [40].

A person's decision to participate in a programme was influenced by self‐perception of their ability to take part which in turn is influenced by personal experience. Being confident in their ability to participate is a facilitator and strengthened by a straightforward, low‐hazard programme [42]. An unacceptable emotional burden associated with the programme was a barrier [42]. Positive personal experiences acted as a facilitator. Those engaging in modern HCV treatments often found them easy to take, with positive clinical and nonclinical outcomes, leading to continued involvement [26]. However, negative personal experiences, usually based on older interferon‐based regimes, were a barrier to participation. The side effects of older treatments led to perceived personal weakness and potential vulnerability within the custodial setting [21].

Competing priorities such as comorbidities, substance use, coping with the stress of incarceration or family commitments may take precedence over treatment for some individuals [53]. For others, programme participation was a priority since they valued the benefits which improving their own health would bring to their family [21].

Incarceration involves punishment through the removal of liberties and the right to refuse healthcare can be one of the few freedoms available. The PIP may decide to not participate in preventative or nonurgent healthcare, despite negative long‐term consequences, for the current opportunity of expressing choice [30].

**Synthesised Finding 3: Social interactions and relationships both within the community of PIP and between them and staff groups influence participation in viral hepatitis programmes**.

This synthesised finding is underpinned by four categories, developed from 21 findings taken from interviews with PIP, six from healthcare staff and four from custodial staff (Table 4; Appendix S5). This finding explains how the social interactions and relationships both within the community of PIP and between them and staff groups influence intervention participation. It demonstrates the influence of social capital, trust and respect, enacted stigma and duty of care.

**TABLE 4:** Synthesised Finding 3.

Synthesised Finding 3	Categories	Example illustrations
Social interactions and relationships both within the community of people in prison and between them and staff groups influence participation in hepatitis programmes	1: Social capital can be a facilitator or barrier.	‘It took a long time for me to convince him to do the treatment too. “(Why did you convince him?) Because he's my mate”’. (PIP) [38]. p4 ‘Still people are going to share [syringes] … rather than coming over to get a new one, if they haven't got one there and there's a shot sitting in front of them and there's three persons there, they're not going to run up and get a new syringe, they'll just use that one’. (PIP) [35]. p504
	2: Trust and respect are facilitators to intervention participation.	‘Nurses build great relationships with prisoners and you see the nurses up there, they are second to none. They are brilliant … She gave me some amount of help up there. They are terrific and have a great rapport and a great respect’. (PIP) [26]. p8 ‘Like more on the public health nurse, yeah, I'd probably trust the most. […] Probably ‘cause […] I've got a good relationship with her. […] She seemed genuine that she cared and that, unlike, you know, most of the screws [corrections officers] from around here—they don't give a shit’. (PIP) [48]. p249
	3: Within the prison setting, enacted stigma was a barrier to interventions.	‘You have to be careful around people, they may not treat you the same’. (PIP) [45]. p216 ‘That's why I got sacked from the kitchen because I had the virus… Pure ignorance to take me out of the kitchen, just because I had Hepatitis C and at that stage it was gone’. (PIP) [23]. p4 ‘It's part of your [prison nurse] job to deal with it [hep C] you know what I mean? Part of your job not to criticise us for having hep C or stuff like that, like if I'm out [in the community] and I go to a clinic, I feel like they are criticising me whether or not they are, but like either way, that's just the way I feel so I won't even bother’. (PIP) [42]. p1156
	4: Duty of care caused conflict.	‘There are security issues though [with security staff at health interviews]; I would not like to do it without an officer … I feel conflicted’. (HS) [25] p4. ‘There's a lad, got a set of hair clippers and he'll lend it to two or three others on the wing while he had hepatitis, and you just feel like going up to them and giving them a nudge and saying “listen you really shouldn't be using those shears,” but we can't’. (CS) [29]. p8

Social capital embodies the ‘features of social organization such as networks, norms and social trust that facilitate coordination and cooperation for mutual benefit’ [54]. This review found the concept of bonding social capital captures the beneficial feelings a PIP gains by being part of a group and wanting to conform or behave like others in the group. This bond served as a facilitator when the group promoted a programme or participation strengthened friendship [38] but was a barrier when strong bonds within prison drug‐injecting networks promoted continued use of nonsterile equipment [35]. Trust and respect within a relationship can also be utilised for effective therapeutic encounters either between PIP and healthcare staff or between PIP and fellow peer educators [48, 51]. Discrimination resulting from HCV's association with injecting drug use led to enacted stigma, subsequent confidentiality concerns and reduced programme participation [45]. Conversely, nonjudgemental interactions promoted programme participation [42].

Without explicit protocols, social bonds within staff groups and between them and PIP caused duty of care conflicts which led to altered programme participation. Staff experienced conflict between maintaining patient confidentiality and reducing a security or infection risk when security staff were present at health interviews [25] or when blood contact occurred [29].

**Synthesised Finding 4: The organisational structure of the prison and healthcare services within it affect participation in viral hepatitis programmes**.

The finding was synthesised from five categories comprising 27 findings from interviews with PIP, 19 from healthcare staff and 11 from custodial staff (Table 5; Appendix S5). This synthesised finding incorporates how the organisational structure of the prison and healthcare services within it affect programme participation. Complementary custodial and healthcare systems facilitate passage through a viral hepatitis care pathway, increasing participation.

**TABLE 5:** Synthesised Finding 4.

Synthesised Finding 4	Categories	Example illustrations
The organisational structure of the prison and healthcare services within it affect participation in hepatitis programmes.	1: The stability of prison life served as a facilitator.	‘Hard enough for us to cope as it is outside with everyday life without throwing that on top. The opportunity to do it in prison you don't have all the stresses of life to go with it, you're more willing to take it on’. (PIP) [23]. p5
	2: Security requirements impact healthcare access.	‘Security comes first and we may never get to see the prisoners’. (HS) [25]. p3 ‘You can't treat'em till we get security right. You just cannot’. (CS) [29]. p6
	3: The availability and organisation of prison healthcare can facilitate or obstruct programme access.	‘Anything that stops us going outside the main gate is good’. (CS) [24]. p6 ‘Certainly, the awareness of the Hep C has gone up in the last couple of months with the fibroscanner [on‐site]… I see more engagement with staff and prisoners’. (CS) [24]. p6 ‘I wasn't there long enough to get the treatment. So they wasn't offering [it to] me. I was only there for 90 days’. (PIP) [32]. p4 ‘It is very urban centric and then you get out of the urban areas … more rural … and we are not as invested in it and it's not as high profile’. (HS) [25]. p5
	4: The timing of the intervention during the prison stay influenced uptake.	‘Make it automatic when you come in on committal’. (PIP) [23]. p5 ‘A big group to do everyone there and then people are coming in with withdrawals… it's a difficult situation for us to be in too’. (PIP) [23]. p5
	5: Limited custodial and healthcare resources restricted programme access.	‘The program generally focuses on group therapy activities because they don't have the structure or sufficient facilities and staff to provide individual services’. (PIP) [48]. p357 ‘Insufficient prison staff restricts movement to healthcare’ (PIP) [42]. p1156 ‘Well, the fact that – because the University is paying for this, that's a major—because we, if we don't have enough officers, we'll have posts like stripped or closed down whereas because this is run by the University, so they're actually paying for our time. […] Even if we're 20 [officers] short, they can't redirect me because I'm not under Corrective Services guidelines today, like I'm at the University's disposal, so that's been good in ensuring that it does continue to happen because that is a big issue’. (CS) [40]. p3 ‘In prison …, I'm not lying you want to see the state of the works ((syringes and needles)) …. The spikes ((needles)) …do be bent … about six people like using them. There's about 30 people on a landing and I'd say between the three landings there's only about four or five syringes… and half of them on the landing would be using the syringes, you know? very dangerous’. (PIP) [44]. p143

The routines of imprisonment gave relative stability to PIP who led chaotic, stressful lives outside of prison, affording them time and motivation to address health issues [23]. Prison routines became a barrier to healthcare when the priorities of each conflicted. Security requirements always take precedence over healthcare, so programme access only occurs if security is controlled [25, 29].

The organisation of prison healthcare also influenced programme access. Streamlined, on‐site care improved pathway navigation, increased awareness and removed barriers such as escorted hospital visits [24]. Poor linkage of care between prison sites, and with outside prison services, interrupted care pathways for the often‐transient prison population [25, 32]. Healthcare access was obstructed by restrictive application procedures or inconsistent availability, both within and between facilities [25, 33, 50].

The optimum timing for programme participation was dependent on the routines of the individual facility and cooperation between custodial and healthcare services. Reduction programmes initiated at integrated prison admission healthcare were viewed as routine and less stigmatising [23]. However, the prison admission process at some facilities can be stressful for PIP with programme uptake improved by delayed scheduling [23, 30].

Limited custodial and healthcare resources restricted programme access, through limited staff or programme availability. For example, when insufficient staff were available for individualised healthcare [48] or to enable prisoner movement [42], scarcity of harm reduction measures led to continued unsafe injecting drug use and treatment unavailable due to funding restrictions [25, 48].

### 3.4 Confidence in the Review Findings

Confidence in the review findings (as assessed by ConQual) was moderate for Synthesised Findings 1, 2 and 3, relating to knowledge, personal views and social interactions and low for Synthesised Finding 4, relating to organisational structure as detailed in Table 6.

**TABLE 6:** ConQual summary of findings.

Title: A Qualitative Systematic Review of Barriers and Facilitators to Hepatitis B and C Programmes in Prisons					
Synthesised findings	Type of research	Dependability	Credibility	ConQual score	Comments
Accurate, up‐to‐date knowledge of hepatitis disease and treatment among people in prison and staff is a facilitator to uptake in hepatitis programmes, particularly when imparted by a trusted source.	Qualitative	Downgrade one level (−1)^a^ (16/31 findings from studies with moderate or low dependability)	Unchanged^b^ U = 24, C = 7	Moderate	31 findings from 18 studies
2 Personal subjective and relative views have a bearing on hepatitis programme participation.	Qualitative	Downgrade one level (−1)^a^ (16/36 findings from studies of moderate or low dependability)	Unchanged^b^ U = 30, C = 6	Moderate	36 findings from 15 studies
3 Social interactions and relationships both within the community of people in prison and between them and staff groups influence participation in hepatitis programmes.	Qualitative	Downgrade one level (−1)^a^ (19/33 findings from studies of moderate or low dependability)	Unchanged^b^ U = 26, C = 7	Moderate	33 findings from 19 studies
4 The organisational structure of the prison and healthcare services within it affects participation in hepatitis programmes.	Qualitative	Downgrade one level (−1)^a^ (17/34 findings from studies of moderate or low dependability)	Downgrade one level (−1)^c^ U = 23, C = 11	Low	34 findings from 14 studies

^a^ Downgraded one level due to common dependability issues across a significant number of included studies.

^b^ Unchanged as synthesised finding is underpinned by a high number of unequivocal (U) findings across all categories.

^c^ Downgraded one level due to significant number of credible (C) findings across all categories.

*Note:* Study dependability: High = 4–5 yes responses to dependability criteria, moderate = 2–3 yes responses and low = 0–1 yes responses.

## 4 Discussion

This systematic review articulates four synthesised findings which identify and explain facilitators and barriers to HBV and HCV programmes in prisons. This discussion reflects on how these findings relate to previous work and how this knowledge may be utilised to enhance programme implementation.

### 4.1 The Importance of Knowledge

This review found that knowledge of viral hepatitis disease, the potential associated health harms and possible treatments among PIP and staff promotes programme uptake with inaccurate knowledge serving as a barrier. This shares similarities with recent reviews exploring hepatitis programme uptake in community populations at risk of HCV and during the transition away from custodial settings [55, 56].

The accuracy of information and who imparts it are important considerations. Newer, streamlined treatment programmes with reduced side effects became available from 2011 [7]. Without formal training, imparted knowledge is potentially out‐of‐date, describing more severe consequences than currently occur. PIP are known to frequently acquire health knowledge from other incarcerated individuals so they should receive viral hepatitis education in addition to health and custodial staff [55]. Formal peer education programmes have been utilised to impart many types of knowledge within prison systems [57] and two included studies reported on such schemes [48, 51]. A systematic review of formal prison peer education has found it to be effective [57] although it is of interest that studies in our review showed informal peer knowledge transfer to have more mixed outcomes [24, 30, 33]. The use of peer educators has raised confidentiality concerns [26] which could be mitigated by formal oversight of these schemes. The timing, format and content of educational materials for PIP were also raised as important for knowledge transfer and different to community environments, with consideration of literacy level and potential consequences of being seen to engage with education required [50].

The findings of this review suggest a need for widespread availability of accurate, up‐to‐date knowledge of HBV and HCV (how they are acquired, health implications and testing and treatment regimens) for PIP and all prison staff. The information must be accessible, in appropriate language and format and available in a nonstigmatising manner. The information should be imparted by a trusted source. If peer education is used, it should be a formal, monitored programme.

### 4.2 Personal Subjective and Relative Views

The personal subjective and relative views of the PIP have a bearing on programme participation. These are multifaceted with each having the potential to be either a barrier or facilitator, depending on the individual's own perceptions and past experiences. Prison demographics may give broad generalisations of characteristics from which associations are applied but a person's reason for accepting or denying a programme will be personal to them. A person's imported value system and influences prior to prison entry continue to shape their behaviour once incarcerated [58].

Globally, at least one in five PIP is detained for illegal drug use [8]. A previous review exploring experiences of HCV testing and diagnosis among people who inject drugs in the community drew conclusions comparable to this synthesised theme, with programme participation being dependent on shifting priorities, lifestyle chaos level and personal risk perception [59]. While incarceration can bring stability and aid participation, an individual's unique motivations should be recognised within the wider programme framework.

The findings of this review suggest that any HBV or HCV programme framework includes some flexibility to allow recognition of the individual and a degree of personalised care. Awareness of the significance of PIP personal views on participation should be incorporated into custodial and healthcare staff training.

### 4.3 Social Interactions and Relationships

The social interactions and relationships both within the community of PIP and between them and staff groups can be either a facilitator or barrier to HBV or HCV programme participation. Prisons are complex environments with multiple interwoven relationships and influences which can be difficult to separate [58]. This review found the concepts of social capital and trust underpinned successful healthcare interactions, therefore focusing on these within viral hepatitis programmes will enhance participation.

The sociological concept of ‘prisonization’ incorporates the impact of PIP taking on ‘the general culture of the penitentiary’ and adhering to the ‘inmates code of behaviour’ [15]. While this can lead to a contraculture among PIP [58], knowledge of its influence, intertwined with social capital, can be utilised for the benefit of healthcare. HBV and HCV programme participation will be facilitated if it is a feature of normative behaviour within the environment.

Trust is an essential component of effective therapeutic encounters [60] and has been shown to facilitate engagement with HCV care in prison settings [34]. This was a frequent concept within included papers but despite its significance to PIP, mistrust was pervasive within the environment [46].

The findings of this review suggest that therapeutic relationships within viral hepatitis programmes are built from a foundation of trust and respect. Programme participation should be integrated into normative behaviour to minimise stigmatising actions.

### 4.4 Organisational Structures of Prison and Healthcare Services

The organisational structure of the prison and the healthcare services within it affect participation in HBV and HCV programmes. Complimentary organisational structures facilitate passage through the viral hepatitis care pathway.

The United Nations has minimum rules for the treatment of PIP which includes the state being responsible for access to the same standard of healthcare as available in the community [61]. The predominant function of prisons to punish individuals and protect society from harm can conflict with healthcare provision and reduce the services available. Proven harm reduction measures such as needle exchange programmes are particularly lacking in prison environments [62]. The different management structures, funding and outcome goals of prison healthcare and security within and between prisons of the same country also contribute to organisational conflict and reduce programme uptake [25].

Ease of movement through the care pathway also influences programme involvement. Community studies have demonstrated the importance of a streamlined care pathway to improving hepatitis programme uptake [57] which was borne out in this review. Point‐of‐care testing [22] and healthcare telementoring [49] are examples of facilitators via process streamlining. Continued focus on the complete care pathway is required to tackle the risk of HCV transmission due to care interruption from frequent prisoner movement [63].

The findings of this review reinforce the importance of collaboration between custodial and healthcare services to build streamlined care pathways which are appropriate to the facility and population and integrate with community services.

### 4.5 Strengths and Limitations

Previous reviews have focused on specific aspects of hepatitis interventions in prisons, such as testing and treatment uptake or linkage to community care [39, 55, 59, 64, 65] and incorated few studies of modern HCV treatments [55, 64, 65]. This review has expanded on current knowledge by adopting a comprehensive search across all HBV and HCV care‐related interventions, enabling a broad review focus which we believe enhances the relevance and transferability of the findings to the diverse range of custodial hepatitis programmes available.

This review has brought together the experiences of PIP, healthcare and custodial staff. Almost two‐thirds of findings were from experiences of PIP, with a fifth or less being from each staff group. The findings were synthesised together due to their similarities and the multidimensional perspective this will give to the conclusions, but we acknowledge the depth of experience expressed within each group is reduced.

This review is limited by the included studies all being from high‐income countries and reflecting their prison and healthcare systems. Prison systems vary greatly across the globe. Those within the global south are poorly resourced in comparison to the north with austere, punitive regimes and minimal health promotion [66, 67]. The people incarcerated generally have little input from custodial staff, often organising their own daily routines and discipline with encounters underpinned by negotiation more than conflict [66]. Further research is required in diverse locations, particularly sub‐Saharan Africa where custodial health promotion is atypical [67], to assess the relevance of these findings to more diverse prison regimes.

The findings are limited by potential bias among the sampled populations of included studies. Experiences of men and women are included but reporting of ethnicity when it occurred was varied. The difficulties with researchers gaining access to conduct prison studies meant that included studies frequently employed purposive sampling and PIP deemed to pose a risk to researchers were excluded. Therefore, the findings may not reflect the complete prison population.

## 5 Conclusion

The higher proportionate prevalence of HBV and HCV infections within prisons and the lower uptake of viral hepatitis reduction programmes compared to the community has led to a focus on the participant's experience within these settings. Prisons are heterogeneous complex environments, and their health programmes should be tailored to the needs of the individual prison and the population it serves. This review has identified four themes concerning facilitators and barriers to prison HBV or HCV programmes and provided recommendations to enhance uptake with the aim of reducing the hepatitis burden within prison environments.

## Conflicts of Interest

The authors declare no conflicts of interest.

## Supporting information


Appendix S1:

Appendix S2:

Appendix S3:

Appendix S4:

Appendix S5:

## Data Availability

The data that supports the findings of this study are available in the Supporting Information of this article.
