# Supplementary material for: A Qualitative Systematic Review of Barriers and Facilitators to Hepatitis B and C Programmes in Prisons
Source: J Viral Hepat. 2024 Dec 28;32(2):e14049. doi: 10.1111/jvh.14049 (PMC11681497; doi:10.1111/jvh.14049)
Supplement: Supplementary file 1 — Appendix S1: [file JVH-32-0-s001.docx]

**A Qualitative Systematic Review of Barriers and Facilitators to Hepatitis B and C Programmes in Prisons**

**S1: Search Strategy**

**Ovid Medline**

1. exp Prisons/
2. (prison* or (correction* adj2 (institution* or centre* or center* or facilit*)) or jail* or gaol* or (detention* adj2 (centre* or center* or facilit*)) or inmate*).mp.
3. 1 or 2
4. exp Hepatitis/ or exp Hepatitis, Chronic/ or exp Hepatitis Viruses/ or exp Hepatitis, Viral, Human/ or exp Hepatitis B/ or exp Hepatitis C/ or Hepatitis B virus/ or exp Hepacivirus/ or exp Viral Hepatitis Vaccines/
5. (hepatitis or (Hep* adj2 (B or C)) or HBV or HCV or HepB or HepC).mp.
6. 4 or 5
7. 3 and 6
8. focus groups/
9. interviews as topic/
10. narration/ or qualitative research/
11. ((face or f2f or “in person” or guided or depth or indepth or informal or “semi-structured” or semistructured or structured or unstructured) adj4 (discussion* or interview* or questionnaire*)).ti,ab,kf.
12. (ethnograph* or (field adj1 work) or fieldwork or (focus adj1 (group or groups)) or (key adj1 (informant or informants)) or qualitative).ti,ab,kf.
13. 8 or 9 or 10 or 11 or 12
14. 7 and 13
15. limit 14 to yr=”2010-Current”
