# Supplementary material for: A Qualitative Systematic Review of Barriers and Facilitators to Hepatitis B and C Programmes in Prisons
Source: J Viral Hepat. 2024 Dec 28;32(2):e14049. doi: 10.1111/jvh.14049 (PMC11681497; doi:10.1111/jvh.14049)
Supplement: Supplementary file 2 — Appendix S2: [file JVH-32-0-s004.docx]

S2: Blank data extraction form

| **Lead  Author** | **Year** | **Title** | **Country** | **Setting** | **Methodology** | **Method** | **Participants** | **Data collection  period** | **Phenomena of  interest** | **Intervention  studied** | **Authors conclusions** |
| --- | --- | --- | --- | --- | --- | --- | --- | --- | --- | --- | --- |
|  |  |  |  |  |  |  |  |  |  |  |  |
|  |  |  |  |  |  |  |  |  |  |  |  |

| 1. Is there congruity between the stated philosophical perspective and the research methodology? | 2. Is there congruity between the research methodology and the research question or objectives? | 3. Is there congruity between the research methodology and the methods used to collect data? | 4. Is there congruity between the research methodology and the representation and analysis of data? | 5. Is there congruity between the research methodology and the interpretation of results? | 6. Is there a statement locating the researcher culturally or theoretically? | 7. Is the influence of the researcher on the research, and vice-versa, addressed? | 8. Are the participants, and their voices, adequately represented? | 9. Is the research ethical according to current criteria or, for recent studies and is there evidence of ethical approval by an appropriate body? | 10. Do the conclusions drawn in the research report flow from the analysis, or interpretation of the data? | **Critical appraisal total** | **General comments** |
| --- | --- | --- | --- | --- | --- | --- | --- | --- | --- | --- | --- |
|  |  |  |  |  |  |  |  |  |  |  |  |
|  |  |  |  |  |  |  |  |  |  |  |  |
