# Supplementary material for: A Qualitative Systematic Review of Barriers and Facilitators to Hepatitis B and C Programmes in Prisons
Source: J Viral Hepat. 2024 Dec 28;32(2):e14049. doi: 10.1111/jvh.14049 (PMC11681497; doi:10.1111/jvh.14049)
Supplement: Supplementary file 3 — Appendix S3: [file JVH-32-0-s005.docx]

References from databases/registers **(n = 662)**

Embase (n = 335)

MEDLINE (n = 166)

CINAHL (n = 101)

PsycINFO (n = 60)

References from other sources **(n = 1)**

Citation searching (n = 1)

Grey literature (n = 0)

**Identification**

Studies included in review **(n = 28)**

Studies excluded **(n = 362)**

Studies not retrieved **(n = 0)**

Studies assessed for eligibility **(n = 45)**

Studies sought for retrieval **(n = 45)**

Studies screened **(n = 407)**

Studies excluded **(n = 17)**

Wrong context (n = 1)

Wrong phenomena of interest (n = 7)

Wrong study design (n = 8)

Wrong patient population (n = 1)

References removed **(n = 256)**

Duplicates identified manually (n = 12)

Duplicates identified by Covidence (n = 244)

**Screening**

**Included**
