# Supplementary material for: A Qualitative Systematic Review of Barriers and Facilitators to Hepatitis B and C Programmes in Prisons
Source: J Viral Hepat. 2024 Dec 28;32(2):e14049. doi: 10.1111/jvh.14049 (PMC11681497; doi:10.1111/jvh.14049)
Supplement: Supplementary file 4 — Appendix S4: [file JVH-32-0-s002.docx]

S4: Critical appraisal of included studies

| Included studies  (Lead author, publication date, reference) | Q1 | Q2^†^ | Q3^†^ | Q4^†^ | Q5 | Q6^†^ | Q7^†^ | Q8 | Q9 | Q10 | Total |
| --- | --- | --- | --- | --- | --- | --- | --- | --- | --- | --- | --- |
| Akiyama 2020^21^ | Y | Y | Y | Y | Y | Y | Y | Y | Y | Y | 10 |
| Byrne 2023^22^ | Y | Y | Y | Y | Y | N | N | N | Y | Y | 7 |
| Crowley 2018^23^ | Y | Y | Y | Y | Y | N | N | Y | Y | Y | 8 |
| Crowley 2018^24^ | Y | Y | Y | Y | Y | N | N | Y | Y | Y | 8 |
| Crowley 2019^25^ | Y | Y | Y | Y | Y | Y | N | Y | Y | Y | 9 |
| Crowley 2019^26^ | Y | Y | Y | Y | Y | Y | N | Y | Y | Y | 9 |
| Dyer 2009^27^ | Y | Y | Y | Y | Y | Y | N | Y | N | Y | 8 |
| Heinemann 2001^28^ | Y | Y | Y | N | U | N | N | N | Y | U | 5 |
| Jack 2017^29^ | Y | Y | Y | Y | Y | Y | Y | Y | Y | Y | 10 |
| Jack 2020^30^ | Y | Y | Y | Y | Y | Y | Y | Y | Y | Y | 10 |
| Jacob 2000^31^ | U | Y | Y | U | U | N | N | U | U | U | 5 |
| Kamat 2023^32^ | Y | Y | Y | Y | Y | Y | Y | Y | Y | Y | 10 |
| Khaw 2007^33^ | U | Y | Y | Y | Y | U | N | Y | Y | Y | 8 |
| Lafferty 2017^34^ | Y | Y | Y | Y | Y | N | N | Y | Y | Y | 8 |
| Lafferty 2018^35^ & Lafferty 2019^36^ & Rance 2020^37^ ^‡^ | Y | Y | Y | Y | Y | N | N | Y | Y | Y | 8 |
| Lafferty 2020^38^ & Lafferty 2021^39^ ^‡^ | Y | Y | Y | Y | Y | N | Y | Y | Y | Y | 9 |
| Lafferty 2022^40^ & Lafferty 2021^41 ‡^ | Y | Y | Y | Y | Y | U | Y | Y | Y | Y | 9.5 |
| Lafferty 2023^42^ & Lafferty 2022^42 ‡^ | Y | Y | Y | Y | Y | N | N | Y | Y | Y | 8 |
| Long 2004^44^ | Y | Y | Y | Y | Y | Y | U | Y | Y | Y | 9.5 |
| Ly 2018^45^ | Y | Y | U | Y | Y | N | N | Y | Y | Y | 7.5 |
| Miller 2021^46^ | Y | Y | Y | U | U | U | U | Y | Y | Y | 8 |
| Mina 2016^47^ | Y | U | Y | U | U | N | N | Y | Y | Y | 6.5 |
| Munoz-Plaza 2005^48^ | Y | Y | Y | U | U | N | Y | Y | Y | Y | 8 |
| Neuhaus 2018^49^ | Y | Y | Y | U | Y | N | N | Y | Y | Y | 7.5 |
| Rehman 2004^50^ | Y | Y | Y | Y | Y | U | N | U | Y | Y | 8 |
| Thornton 2018^51^ | Y | Y | Y | Y | Y | U | N | Y | Y | Y | 8.5 |
| Wurcel 2021^52^ | Y | Y | Y | Y | Y | Y | Y | Y | Y | Y | 10 |
| Yap 2014^53^ | Y | Y | Y | Y | Y | N | N | Y | Y | Y | 8 |

^†^ ConQual dependability questions:

N, No=0; U, Unclear=0.5; Y, Yes=1.

Criteria for the critical appraisal of qualitative evidence:

Q1=Is there congruity between the stated philosophical perspective and the research methodology?

Q2=Is there congruity between the research methodology and the research question or objectives?

Q3=Is there congruity between the research methodology and the methods used to collect data?

Q4=Is there congruity between the research methodology and the representation and analysis of data?

Q5=Is there congruity between the research methodology and the interpretation of results?

Q6= Is there a statement locating the researcher culturally or theoretically?

Q7=Is the influence of the researcher on the research and vice-versa addressed?

Q8=Are participants, and their voices, adequately represented?

Q9=Is the research ethical according to current criteria or, for recent studies, and is there evidence of ethical approval by an appropriate body?

Q10=Do the conclusions drawn in the research report flow from the analysis or interpretation of the data?

^‡^ Findings from the same interview data were reported across multiple papers and treated as one study for this review.
