# Supplementary material for: A Qualitative Systematic Review of Barriers and Facilitators to Hepatitis B and C Programmes in Prisons
Source: J Viral Hepat. 2024 Dec 28;32(2):e14049. doi: 10.1111/jvh.14049 (PMC11681497; doi:10.1111/jvh.14049)
Supplement: Supplementary file 5 — Appendix S5: [file JVH-32-0-s003.docx]

S5: Synthesis of unequivocal and credible findings with illustrations

| **Synthesised finding** | **Category** | **Sub category** | **Lead author, date, reference** | **Finding** | **Finding explanation** | **Illustration** (with page number) | **Credibility** |
| --- | --- | --- | --- | --- | --- | --- | --- |
|  |  |  |  | Black = PIP,  Green = Healthcare,  Blue = Custodial staff |  |  | U = unequivocal C = credible |
| **1. Accurate up-to-date knowledge of hepatitis disease and treatment amongst people in prison and staff is a facilitator to uptake in hepatitis programmes, particularly when imparted by a trusted source.** | 1. Poor prisoner awareness of hepatitis disease testing and  treatment availability. |  | Rehman 2004^40^ | The need for more information | Many concerns were raised regarding the type,  quantity, and quality of the information the prisoners were receiving. | Well, there’s RAPS and there’s CHIPS. I know that a lot of the stuff that’s told in CHIPS and RAPS . . . There’s information, but there’s not that much. There’s also not much of anything for the effects. Like, you know, how it is [for] somebody getting tested for HIV or Hep C and it come[es] back negative . . . There’s nothing on counselling, what you went through during the time you're waiting for it.' _p65_ | U |
| U = 24 C = 7,  18 studies  ( 8 scored 3 or less out of 5 on dependability) |  |  | Yap 2014 ^43^ | Knowledge | Lack of awareness of HCV treatment in prison was a barrier. | Only after a period of incarceration ( weeks to months or even years) did they learn from other inmates or a nurse that treatment was available. One inmate was unsure if the treatment was safe or not while another was misinformed about the duration of treatment. _p4_ | U |
|  |  |  | Wurcel 2021^42^ | Knowledge | Knowledge about HCV testing opportunities in jails varied. | I don't think I've ever been offered testing. It's kind of weird that no one has asked. I probably should just for the hell of it … It would be good to know.' _p4_ One said he wouldn't be interested because he needed to learn more about it. 'They need to tell me "it's for this and that". They need to tell me about the complications.' _p4_ 'I heard [HCV] was something I don't want, I was told it was an STD, but honestly I don't know. I hear a lot of shit about shit and don't believe it.' _p5_ | U |
|  |  |  | Ly 2018^45^ | HCV knowledge | HCV knowledge was generally low | Nearly a third of the participants had never heard of HCV. Misunderstanding of HCV risk was common. 'I know it is a deadly disease, Hepatitis C got lots of germs. If you bathe in your own bodily fluid, then it give you Hepatitis C!' _p216_ | C |
|  |  |  | Dyer 2009^27^ | Lack of knowledge | Prisoners had received a pamphlet but had little understanding of what their positive diagnosis meant. Disposable razors and toothbrushes were reported to be available, although possibly not always accessed. | I thought it meant I was going to die.' _p39_ 'It is common for uninformed prisoners to share personal equipment to reduce costs. Many avoid prison tattoos when they are aware of the risks.' _p39_ | U |
|  |  |  | Crowley 2019^25^ | Prisoners' lack of knowledge and fear of treatment | Lack of knowledge among prisoners was seen as a barrier to engagement with HCV care, also linked with fear of treatment including liver biopsy and interferon based treatment regimes. | They (prisoners) can be the biggest block not because they are opposed to it but because they don't have the knowledge.' _p4_ | U |
|  | 2. Low intervention uptake due to poor staff knowledge. |  | Munoz-Plaza 2005^39^ | Lack of knowledge | Staff members lack knowledge about HCV. | [There is] very low interest in hepatitis, probably because they [staff] don't know much about it and it doesn't have the hype of HIV.' _p362_ | C |
|  |  |  | Mina 2016^47^ | Prisoners judged as unsuitable for assessment by the healthcare team | The healthcare team may judge the prisoner to be unsuitable for assessment and treatment due to their physical and mental health or ongoing IDU. | There is poor education and awareness, and a lot of inexperience regarding managing complex health issues (such as hepatitis C).' _p6_ | C |
|  |  |  | Byrne 2023^46^ | Individual knowledge | Existing knowledge of the test or prior  experience of point of care testing were facilitators. | Clinical staff found it easier to navigate the prison  environment for testing after being 'key trained'. _P8_ A lack of awareness of HCV among people in prison and prison staff hindered improvements to prison care. _P7_ Staff found it easier to implement the GeneXpert pathway because of previous testing undertaken in the prison for diabetes by another team. _p8_ | U |
|  |  |  | Crowley 2019^25^ | Skillset and concerns regarding phlebotomy | Nurses of the Prison Healthcare Service had a specific  skill set that matched the complex health needs of prisoners. Some had a fear of phlebotomy linked to known high levels of BBV infection among prisoners. | There's a particular skill set that is prison nursing … knows the care plan of that individual, knows the dynamic of the environment and can support the patient through that treatment.' _p3_ 'There's a problem of skill mix. Lot's of nurses don't take bloods. If you have nobody trained up that's a problem. Some nurses are afraid to take bloods. There's a fear. The virus itself ... people not confident taking bloods.' _p3_ | U |
|  |  |  | Jack 2017^28^ | Education | All prison custody officers knew that HCV is transmited via sharing drug injecting paraphernalia but were less clear about other modes of transmission and the consequences of infection. | If their [prison officer] understanding was a little better,  perhaps the panic wouldn't be so bad, y'know 'I've got a bit of blood on my skin, oh Christ I must have it now' and y'know phone calls up to healthcare, every single ailment 'oh my God, oh my God, I'm not sleeping.' _p10_ | U |
|  |  |  | Crowley 2018^26^ | Lack of knowledge | All grades of staff felt their lack of knowledge in relation to the newer HCV treatment and risks of transmission impacted on their ability to engage with prisoners on this issue. | Staff worry about HIV and HCV … we need more education programs and training for staff but its also hard to release staff for training.' _p4_ | U |
|  |  |  | Lafferty 2022^35^ | Ethicality | Encompassing personal perspectives of whether or not people in prison were deserving of HCV treatment. | Correctional officers’ opinions of patient’s treatment worthiness  appeared to be intertwined with knowledge of availability of comparable HCV treatment within the community. 'So I was dead against it at first, but now I’m like, “yeah it’s good”. [So what changed?] When [the SToP-C officer] started explaining that it could actually wipe out hepatitis C in the community as well. My initial thing was, “why are these blokes getting [HCV treatment] when they break the law and they’re in jail, when people in the community couldn’t be getting it”, but now they are getting [treatment].' _p4_ | U |
|  | 3. People in prison fear hepatitis due to incorrect knowledge |  | Jack 2020^29^ | Knowledge | Fear and stigmatizing behaviours were provoked by a lack of understanding and knowledge of HCV infection. | In my last prison there was a guy serving people food who had hepatitis and I didn't know this until someone said "I don't want that guy in here, you got hepatitis" and I was like, I don't think he can work on the server, how is he serving me my food if he's got hepatitis?' _p4_ | U |
|  |  |  | Khaw 2007^31^ | Prisoner's fears and lack of knowledge  about HCV | Many were not aware of disease prognosis, treatment options and outcome. Some assumed that a positive diagnosis was a 'death sentence'. | Er, I don't know really, er, I don't really know, I mean, I think like I say, I think people are just frightened ye na [you know]. People are frightened to get the test ye na [you know], thinking that it could be a killer not knowing what, not knowing what it actually is, what it actually does to you, I mean?' _p3_ | U |
|  |  |  | Jack 2020^29^ | Fear | Fear of contracting HCV infection and being fearful of a positive HCV test result. | They might pass it on to them, it's like AIDS, when AIDS first came out, people thought thay can't share a cup with someone, can't touch them, you can't be near them … it's a threat to me.' _p4_  'People are scared, what they don't know don't hurt them. If you told me I've got cancer I'm going to worry for months until I do pop off, that's going to be more torture to me than not knowing.' _p4_ | U |
|  | 4. Fear to start treatment due to poor or out of date knowledge |  | Akiyama 2020^22^ | Variable knowledge of new HCV therapies  affecting attitudes toward HCV treatment | Those aware of DAA were more inclined to start HCV  treatment. Poor knowledge of DAAs led to concerns about adverse effects of treatment. | Yeah. Pills. No injections. I don't want any needles.  I'm done with that…It's just a trigger' _p5_ 'Of course, anytime you start [hepatitis C] treatment. That's like taking chemo for cancer' _p5_ | U |
|  |  |  | Crowley 2019^23^ | Lack of knowledge | Many participants still associated treatment with interferon-based therapies and related side effects. Many described not being aware of the new treatment options. | They say knowledge is power. I didn't know. I thought you had to inject yourself … afraid of the needle buzz and all that, couldn't believe it was only a tablet.' _p5_ | U |
|  |  |  | Crowley 2018^24^ | Lack of knowledge | Lack of prisoners knowledge was a major block to engagement with HCV treatment services. They were often confused about different types of hepatitis and modes of transmission. Misinformation existed among prisoners. | Most people don't have an understanding. You talk to someone who says something then you ask someone else and they'd tell you a completely different story. So, there's misinformation. We still don't know anything about it you know' _p3_ | U |
|  |  |  | Crowley 2019^23^ | Fear of treatment | Many had witnessed friends struggling with historical treatment regimens or had previously discontinued interferon-based treatment due to side effects. | The bleeding nightmares and sweats were really, really bad … was on it for a few … a couple of weeks in 2001 and then I just stopped.' _p5_ | U |
|  |  |  | Crowley 2018^24^ | Fear of treatment and having a liver biopsy | Prisoners' fear of treatment and 'horror stories' they had heard from other inmates. | They used to get the injection but it made her sick or something. It did. She said she was very sick. Some people say I'd rather die than do the treatment'. _p3_ | U |
|  |  |  | Yap 2014^43^ | Treatment related fears | Fears associated with treatment. | Inmates dreaded medical procedures such as liver biopsies, the size of needles associated with interferon injections or feared relapse to injecting drug use as a consequence of injecting during treatment. _P5_ 'Well they find it very hard to get blood from me. Very, very, very hard to take blood from me. They tried three times today and still couldn't get no blood, and ... that was enough for me.' _p5_ | C |
|  |  |  | Mina 2016^47^ | Side effects of treatment | Inmates were worried about the side effects of treatment. They may lose prison work and entitlements. | Prisoners fear the side effects of treatment, they fear they will "lose their mind".' _p8_ | C |
|  |  |  | Crowley 2018^26^ | Prisoners' fear of treatment  and stigma | Fear of treatment was linked to: side effects of treatment, liver biopsy and the concerns about stigma. | In terms of history and patient issues, patients think they're going to get very sick on it (interferon), they think they're going to end up in a heap.' _p5_ | U |
|  | 5. PIP believe information when from a credible source | Credible peer education | Akiyama 2020^22^ | The importance of other incarcerated persons in communicating HCV-related knowledge | Communication with other inmates was a key source  of information for HCV knowledge | I had a roommate not too long ago. He's Hep C and  was telling me the treatment and he said he take medication for it and he said yo, get the treatment. You know what I mean? I said okay. I go get the treatment' _p6_ | U |
|  |  |  | Munoz-Plaza 2005^39^ | Credibility of peer education | Education from fellow inmates was cited as more credible than that from staff. | One inmate said that while he was open to having staff provide education about HCV or other health topics, he believed that many inmates are stubborn about not listening to staff and would be more receptive to peers. _P355_ 'A lot of staff, they know what we do in here, but nobody is going to listen to them, but if its is another guy in blue sits down with him, he'll go, "Oh, now that hits".' _p355_ | U |
|  |  |  | Crowley 2018^24^ | Peer support | Prisoners described trusting peers, particularly those  who had completed HCV treatment. | Yeah from a prisoner to a prisoner. It's not like you're going to be a teacher giving a lecture. You're just sitting down talking about how you catch it and just educating people'. _p5_ | U |
|  |  |  | Crowley 2018^26^ | Peer workers | Trained peer workers had the potential to facilitate prisoner engagement with health services. | Peer led education is better than say something coming from the top down. Let the guys at the ground work up.' _p5_ | U |
|  |  |  | Munoz-Plaza 2005^39^ | Greater access to peer educators | Peer educators have more daily access to prisoners than staff, which results in increased opportunities for informal and confidential conversations about sensitive concerns like HCV. | I believe they listen to their peers more than us .. They are able to talk with them in the dorms during times when we wouldn't have access to them.' _p356_ | C |
|  |  | Trusted source | Miller 2021^38^ | Education from trusted source | Trust is an essential component of effective therapeutic encounters. | Who do you trust about hepatitis C information? 'Probably the clinic. Pamphlets, to a lesser extent. Like more on the public health nurse, yeah, I’d probably trust the most. P249 | U |
|  |  |  | Dyer 2009^27^ | Participation in education programmes | Incentives were offered to promote participation in education programmes, such as certificates, celebrity visits, novel foods not supplied by the prison. | Bribery works' _p38_ | C |
|  |  |  |  |  |  |  |  |
|  |  |  |  |  |  |  |  |
|  |  |  |  |  |  |  |  |
|  |  |  |  |  |  |  |  |
| **2. Self and health beliefs of the person in prison** | 1. Felt stigma. If hepatitis status is known, as associated with drug use, fear they will be thought less of. (Internalised feelings, shame, fear) | Fear they will be thought less of | Lafferty 2018^33^ | Social barrier | Lack of confidentiality and lack of social support were seen as barriers. | They could be feeling like they are in trouble if they were to come up [to the clinic] and try to do something about it [hep C] anyhow, because they'll be thinking everyone else will find out they've got hep C if they are doing the hep C programme and that'll put ... they might not be part of that crowd anymore.' p1529 | U |
| U = 30 C = 6, 15 studies  (7 scored 3 or less out of 5 on dependability) |  |  | Yap 2014^43^ | Social and cultural community factors | Stigma, discrimination and lack of confidentiality influenced willingness to have treatment. | For some, having hepatitis C means you are a junkie so most keep the information private.' _p8_ '… the poor blokes that have gotta do it [treatment] for a year, like you start treatment and by the time you finish it, everyone knows. You know what I mean? ... it may not be big in your eyes but to the person that's actually doing it, it's pretty degrading.' _p8_ | U |
|  |  |  | Munoz-Plaza 2005^39^ | Stigma | There is stigma attached to having HCV infection in  prison. | The stigma can make it difficult for even peer educators to have an impact on some inmates who engage in activities that put them at risk for HCV infection, have HCV but don't know it because they have not been tested or are aware of their HCV positive status but are afraid to do anything about it for fear of discrimination. _p359_ | C |
|  |  |  | Crowley 2019^23^ | Stigma | Feelings of stigma and shame associated with HCV infection, mainly related to its association with IDU. | There should be more education in the prisons about it, for people who don't need it. Because there is a stigma to it, you know what I mean. There's a stigma to someone on gear, but there is a bigger stigma to someone on gear that used needles … that's the way, and it's like, people who use cocaine look down on someone who uses heroin. People who use heroin look down on someone that's injecting heroin. Then people who are injecting heroin with hepatitis c look down on someone who has HIV and it's just mad.' _p7_ | U |
|  |  |  | Crowley 2019^25^ | Stigma | Prisoners' concern around stigma was seen as a block to prisoners engaging in HCV care. | 'You'd see that would be the beauty in universal testing, because then it's expected. It would destigmatise it.' p4 | U |
|  |  | Felt undeserving of treatment | Wurcel 2021^42^ | Stigma and deservingness | Some felt that HCV was not a stigmatized illness but others discussed how stigma was a real barrier to getting tested. The stigma around incarceration impacted whether participants deserved HCV care. | HIV is serious, Hepatitis C isn't as serious.' _p5_ 'In jail they wouldn't treat [HCV] as serious because people look down on inmates ... A white coat will offer more help than an officer, that's my opinion. I'm a human like everyone else.' _p6_ | U |
|  |  |  | Wurcel 2021^42^ | Stigma and deservingness | Some felt that HCV was not a stigmatized illness but others discussed how stigma was a real barrier to getting tested. The stigma around incarceration impacted whether participants deserved HCV care. | Addicts are not, in their mind, good people, they're weak, stupid, evil, mean, selfish … you know all those things. Which, unfortunately, many addicts have heard that so many times, that they start internalising that.' _p5_ | U |
|  | 2. Self-perception of ability to take part in intervention | Feeling positive to take part in intervention | Lafferty 2023^36^ | Affective attitude | How an individual feels about taking part in an intervention. | There was resounding response amongst participants that point-of-care HCV RNA testing provides "quick" results and that participation was "easy". 'No I just, I just like to say like how much of a good process yous have got going for yourselves in here, you know what I mean. Like that was quick, simple and easy.' _p2_ | U |
|  |  | Feeling confident they can participate in the intervention | Lafferty 2023^36^ | Self-efficacy | The participant's confidence that they can perform  the behaviours required to participate in the intervention. | The ease of fingerstick testing was viewed as widely achievable. 'It is a good thing because there are a lot of drug users that do have trouble finding veins that would be in the same boat a me, that would not go and get a blood test, just because of how hard it is to find a vein.' _p4_ | U |
|  |  |  | Thornton 2018^41^ | Self- efficacy, confidence and self- esteem | Peer educators recognized the transformative power of their role. They reported that gaining self‑ esteem and confidence gave them hope that they could take steps towards positive change. | I think it just makes you have a little bit of self‑ esteem. And I think that’s probably one of the biggest problems for someone like me who was addicted to drugs.' _p1551_ | U |
|  |  |  | Akiyama 2020^22^ | Concern for relapse to active drug use and  HCV reinfection | Substance use and relapse were important factors that may deter them from engaging in HCV care. In some cases, this led to the conclusion to avoid HCV treatment currently. | It would be stupid on my part to go through a process like that  and then screw it all up. If I made a decision to do that I would keep being risk free after that.... This is probably why I haven't pursued it so far.' _p7_ | U |
|  |  |  | Lafferty 2022^35^ | Self-efficacy | The participant's confidence that they can perform the behaviour required to participate in the intervention. | Self-efficacy was integral to creating safe and trustworthy pathways for patients to engage in HCV care – from patient retrieval (study officer) and throughout clinical engagement (study nurse).   '[Yeah, so you don’t need to disclose if they report to you that they’ve been injecting drugs?] No. That would be a massive problem if we did that because you can’t get – you can’t do a proper assessment of somebody – so you can’t figure out their risks.' _p5_ | U |
|  |  |  | Lafferty 2022^35^ | Self-efficacy | The participant's confidence that they can perform the behaviour required to participate in the intervention. | Self-efficacy was integral to creating safe and trustworthy pathways for patients to engage in HCV care – from patient retrieval (study officer) and throughout clinical engagement (study nurse). 'See I believe it’s up to an officer in the communication style, to actually be – you know actually communicating with a person, encouraging them to have their needs met, because otherwise like the person can fall through the cracks.' _p5_ | U |
|  |  | Perception of the interventions emotional burden | Lafferty 2023^36^ | Burden | The perceived amount of effort that is required to participate in the intervention. | Point of care HCV RNA testing was viewed as alleviating or mitigating the emotional burden associated with previous testing routines. 'Yeah, it was easy, because you could tell me straightaway you know what I mean , whether … and I wasn't sitting around doing head miles.' _p3_ | U |
|  | 3. Self-perception of their own health and the associated  relevance of hepatitis. |  | Yap 2014^43^ | Individual health | The prisoner’s individual health influenced whether they started treatment. They would not start treatment if they thought their HCV was not severe enough, or treatment was pointless if they were still using drugs. | [How come you're not as interested?] Probably because I feel healthy. I don't know, [..but you said you wanted to do a liver function test.] Yeah, I just wanted to see if it was bad. Because if it is getting real bad then I'll see somebody about it, you know. Maybe get into treatment or whatever. But it isn't really giving me any problems,' _p6_ 'No I've never been on treatment, no. [Would you like to be on treatment?] Yeah, I would but.. I don't want to go through something full-on like that if I'm just going to share with someone again, you know...[And you think you will be sharing with someone again?] Yeah, probably ... I don't want to waste anyone else's time and myself.' _p6_ | U |
|  |  |  | Crowley 2019^23^ | Lack of relevance | Many viewed HCV as benign and lacking relevance and did not attribute any physical or mental health issues to being chronically infected until they had experienced the benefits of treatment. | I was in there (prison) and I got it (BBV screen) done and it was … the best Christmas present I ever got. Like I didn’t even mind I had it … at that stage I was just thinking about HIV.' | U |
|  |  |  | Kamat 2023^30^ | Low perceived risk of harm | The perceived asymptomatic nature of the HCV  disease course led to low motivation to pursue treatment. | It's not visual, I mean, yo don't see the symptoms. It's almost like it's not there … Out of sight out of mind.' _p6_ | U |
|  |  |  | Ly 2018^45^ | Self-perceived risk of infection | Self-perception of low risk was brought up by participants as a reason for not testing in the past. | I have never thought about it (HCV). There were no red flags. With Hepatitis C you get these yellow spots and I don't have any.' _p216_ | C |
|  |  |  | Lafferty 2023^36^ | Identification of candidacy | A person's capacity to self-identify as eligible for  receiving healthcare as well as identification of  health need requiring care. | I don't want to be tested for hep C, because I'm on remand, that sounds stupid.' _p1155_ | C |
|  |  |  | Crowley 2019^23^ | Fatalism | A fatalistic view of their illness (hepatitis) and continued high-risk drug taking based on this view. | The only reason why I kept on using needles is because I said to myself that in my head, I had it. I had hepatitis anyway, so it's not going to make a difference, yo know what I mean. I'm going to die - I thought I was going to die from hepatitis, because my sister died from hepatitis.' _p6_ | U |
|  |  |  | Lafferty 2018^33^ | Structural barrier | The high prevalence of HCV among prisoner populations was seen as a barrier to treatment due to the risk of reinfection. | Outside is better … Because girls who have undergone treatment in here, they're going to keep using aren't they, so they are going to catch it again … you won't get re-infected outside beause you've got the needles, the cleaner needles.' _p1529_ | U |
|  |  |  | Lafferty 2020^34^ | HCV treatment is only half the package | There were concerns about feasibility of self-protection against future risk amid the current policy and structural frameworks with regards to management and treatment options for drug dependence available to incarcerated people. | This created tension for participants who currently injected drugs as they felt caught between two policies priorities: curing HCV, but with no provision for support to protect against re-infection. _P4_ 'Yeah who do the [HCV] treatment, like I guess what they say is, "we'll do the treatment, but there's no use really doing the treatment, because we're just going to continue to shoot up" and you know there's no rehabilitation in this jail or in pretty much in any jail. And you want help to get off whatever you're doing, but they won't put you on methadone or bupe or nothing like that.' _p4_ | U |
|  |  |  | Crowley 2018^26^ | Concerns about personal risk | This concern covered the areas of personal safety and risk of exposure to and acquisition of blood borne viruses. | I worry about myself … with the increased violence actually …, we are exposed more and more to open wounds and stuff.' _p4_ | U |
|  |  |  | Lafferty 2022^35^ | Affective attitude | How an individual feels about their workplace  (i.e., the respective prison) taking part in a health intervention aimed to reduce HCV transmission and prevalence among people in prison. | One of the biggest personal benefits highlighted was the reduced risk  of HCV exposure through either needlestick injury or physical altercation due to reduced HCV prevalence resulting from prison-wide treatment scale up.  'Look [hep C treatment as prevention is] good for the jail. Like I think if you can keep hep C out, you’re going to stop worries about needlestick injuries here […]. Hep C not being in the jail, not being prevalent, is good for so many different reasons. […] I’m talking you know, if you do get a needlestick and hep C’s not there. _p3_ | U |
|  | 4. Engagement with an intervention is dependent on personal experience. | Own experience of intervention | Crowley 2012^23^ | Positive experience of treatment. | Those engaging in or completing treatment found DAA therapies easy to take, tolerable with minimal side effects. | A doddle, flew through it … way easier than expected.' _p8_ | U |
|  |  |  | Crowley 2019^23^ | Transformative clinical and non-clinical changes associated with HCV treatment. | Many participants described positive transformative clinical and non-clinical outcomes from engagement with HCV treatment. | It's like, that was taken away from me. And it's like, there's another chance for it now if you want it, and I've never wanted to get clean as much as I do now, you know what I mean.' _p9_ | U |
|  |  | Fear the intervention will make them vulnerable in prison | Akiyama 2020^22^ | Vulnerability during incarceration and fear  of treatment interruption | Concern for starting treatment in jail. Feelings of vulnerability and physical weakness from treatment would result in an inability to protect themselves while in jail. | The only concern I have is what the treatment entails - if I'm  going to be sick from it; if I'm going to be weak from it. Those are things I need to worry about because I'm in a environment that I can't really depend on right now. I gotta have an awareness that's different from the street.' _p6_ | U |
|  | 5. Competing priorities | Competing priorities | Yap 2014^43^ | Socio-economic considerations | Employment and accommodation may be affected by treatment. Prisoners can work to earn additional income that can be used to pay for luxuries. | One prisoner had delayed her decision for fear of losing her job since treatment would require taking time off work and waiting in the clinic to consult a prison healthcare provider. _P6_ Inmates were reluctant to transfer to another prison offering treatment since they risked losing their preferred accommodation and employment. _p6_ | C |
|  |  |  | Akiyama 2020^22^ | Competing priorities (such as other medical  comorbidities, ongoing substance use and housing | Other priorities (substance use, chronic medical  difficulties and problems outside jail) compete with motivation to seek HCV treatment. | I'm going through so much shit. Right now, I got a little cold. And I know this treatment that it's going to affect me, because my T cells is really low. Not now. I'm not ready for it.' _p7_ | U |
|  |  |  | Crowley 2019^23^ | Competing priorities | The struggle to treat withdrawals and cravings was overwhelming for most and was often the first and most pressing demand. | I was strung out when I came in … They asked me about it (HCV treatment) once or twice but I was, to be honest … my life was chaotic, I couldn't manage it.' _p6_ | U |
|  |  |  | Yap 2014^43^ | Stress | Prisoners were not interested in taking up treatment if stressed by issues deemed to be more important than HCV. | Stress symptoms included sleep disturbance, poor concentration and preoccupation with ongoing problems. _P4_ '… a lot of them don't want to start treatment especially while they're on remand because of the stress of court … They want to be able to present well when they go to court. Also family stresses ... they can be under a certain amount of stress and they don't want any additional stress. _p4_ | U |
|  |  |  | Yap 2014^43^ | Family relationships | Family commitments on the outside may influence treatment decisions. | One refused a transfer to consult a hepatologist since he was concerned about his partner's health and difficulties they would have with transport to reach the (treatment) prison. _P6_ 'A female inmate was concerned with having less energy to care for her young children on the outside after being released and after completing treatment.' _p6_ | C |
|  |  | Motivating priorities | Akiyama 2020^22^ | Social support and the importance of family | Family as a key motivator driving interest in HCV  treatment. | If you know there's a cure…You know, I have five daughters and now I got three grankids. So now I hope - I want to stay alive a little longer. Got some hope…I got grandkids I said…I'd love to be with them._' p8_ | U |
|  |  |  | Thornton 2018^41^ | Sharing information inside and outside prison | Peer educators recognized the ramifications that  sharing knowledge has had on their families and on peers who do not know about HCV or infectious disease transmission. | It’s changed every part of my life. It’s probably changing my kid’s life now, cause, I tell him to wash his hands. It’s not just me, but everyone around me is getting taught.' _p1553_ | U |
|  | 6. Choice as an expression of freedom |  | Jack 2020^29^ | Choice | The deprivation of liberty associated with a prison sentence means that any opportunity to exercise choice and challenge the system is an important feature of prison life. | It's (healthcare) the only freedom that we've got. Something like that (BBV test) if it's an option, and you're trying to force it onto somebody, some of them will just go against it just for the sake of going against it, anti-establishment.' _p5_ | U |
|  |  |  | Ly 2018^45^ | Understanding of testing rights and implicit  coercion. | Most participants demonstrated knowledge of their rights to decline healthcare in jail. However, some participants felt coerced. | I feel like I have to listen and cooperate. If I listen, then it is less stressful. If you have an attitude, then the officers will make it worse for you … I am afraid to say no … I can say no by telling them "next time" .' _p217_ ''I know I can refuse any sort of test ... but if it is required by law like DNA swab and finger printing, then it is accepted.' _p217_ | C |
|  |  |  |  |  |  |  |  |
|  |  |  |  |  |  |  |  |
| **3. Social interactions and relationships influence participation in programmes** | 1. Social capital |  | Lafferty 2017^32^ | Bonding social capital  (connections between inmates) | Connections between inmates (bonding social capital)  provided valuable opportunities for informal dispersal about treatment side effects and was closely linked with HCV treatment awareness, uptake and adherence. It can also have a negative impact on the decision to access treatment. | The only other people I normally talk to about the treatment  are blokes who are on it. Talk about how they’re feeling, how they're coping and just basically just to see if I'm feeling the same things as they are.' _p3_ | U |
| U = 26 C = 7, 19 studies (11 scored 3 or less out of 5 on dependability) |  |  | Lafferty 2020^34^ | Bonding social capital | Bonding social capital (connections between peers) among cellmates, groups within prison yards and injecting networks influenced HCV treatment initiation. | So, my mate that I used to use with, I had to actually tell … what's her name, is it [SToP-C nurse]? I had to give her an instructional thing one day when she wa doing my blood test on how to get a needle into his vein and which vein to hit, because his veins are bloody horrible.[...] It took a long time for me to convince him to do the treatment too. (*Why did you convince him?*) Because he's my mate.' _p4_ | U |
|  |  |  | Lafferty 2018^33^ | Social behaviours around IDU in prisons | The injecting culture in the prison environment was a possible barrier. | Still people are going to share [syringes] … rather than coming over to get a new one, if they haven't got one there and there's a shot sitting in front of them and there's three persons there, they're not going to run up and get a new syringe, they'll just use that one.' _p504_ | U |
|  |  |  | Lafferty 2020^34^ | Prevalence is risk | Prison wide HCV treatment provided assurance that transmission risk (associated with fighting) was greatly reduced. | Like I said, when I had a fight, I was a bit concerned. But with the people I have in my unit now, I know everyone's been treated, so the likelihood of getting reinfected through a fight or something, is very low.' _p4_ | C |
|  |  |  | Lafferty 2023^36^ | Offers and resistance | Non-utilisation of services does not necessarily reflect non-offer and people may decline offers of treatment for a multitude of reasons. | The non-judgemental approach of prison health staff facilitated care management among people entering prison. 'Just the way youse come and approached us and talked to us about it and how everything was worked out and that [the treatment] had a 95% success rate and you know hearing that is like good news you know what I mean.' _p1156_ | C |
|  |  |  | Lafferty 2020^34^ | Linking social capital | Linking social capital (between individuals or groups and institutions) is an important resource for HCV treatment uptake amongst those incarcerated, particularly an ability to trust healthcare personnel within the prison setting. | I freaked out when I found out that I had two different genotypes, like 1a and 3a and I never thought that coud happen you know, so I was a bit worried, but the nurse reassured me and that.' _p5_ '(The SToP-C officer is) good actually. She is good. … She doesn't judge, always has a smile on her face, she tries to help you. She's good. I like her.' _p6_ | U |
|  |  |  | Lafferty 2017^32^ | Linking social capital (connections between inmates and institution) | Linking social capital was a valuable resource for inmates in making decisions about accessing HCV treatment and care. Participants descriptions indicated a degree of trust of clinic staff which in turn enabled participants to express agency and make informed decisions about treatment. | It took [the nurse] a little bit to convince me to do it. And then I  finally done it. And I didn't have no dramas. [But they talked you into it?] They didn't talk me into it, they just explained a few things to me. And you know, give me… didn't push me or anything, just gave me time to think about it and that.' _p3_ | U |
|  |  |  | Crowley 2019^25^ | Peer networks | There were mixed views about the use of peer workers in prison health care. Many had worked with peers in mass screening initiatives and were very positive about their benefits. Concerns were expressed with regard to confidentiality, the accuracy of information, the structures required to ensure governance and maintenance of prison security. | I know peer support can be great but we need to be careful  with the role they can play and that we are not abdicating our role. The governance of it, the security issues and the obligations we have must be considered you know.' _p4_ | U |
|  | 2. Trust and respect are facilitators |  | Crowley 2019^23^ | Positive experience of prison health care | The prisoner's relationship with in-reach hepatology nurses was one of trust, familiarity, support and important in their HCV journey. | Nurses build great relationships with prisoners and you see the nurses up there, they are second to none. They are brilliant … She gave me some amount of help up there. They are terrific and have a great rapport and a great respect.' _p8_ | U |
|  |  |  | Miller 2021^38^ | Trust | Trust is an essential component of effective  therapeutic encounters. | In general, health professionals were seen as trustworthy, while  custodial officers were seen as untrustworthy. In contrast, the trustworthiness of peers was dependent on the individuals and the context. Peers were trusted if they had previous experience with HCV or were considered an ‘elder’ of their group, whereas others were not trusted due to a perceived lack of competence or fidelity. _p247_ Who do you trust about hepatitis C information? Probably the clinic. Pamphlets, to a lesser extent. Like more on the public health nurse, yeah, I’d probably trust the most. [ … ] Probably ‘cause [ … ] I’ve got a good relationship with her. [ … ] She seemed genuine that she cared and that, unlike, you know, most of the screws [corrections officers] from around here - they don’t give a shit. ' _p249_ | U |
|  |  |  | Lafferty 2023^36^ | Perceived effectiveness | The extent to which the intervention is perceived as likely to achieve it's purpose. | It seemed like yous knew what you were doing.' _p3_ *'Did you have any concerns about the accuracy of the test?* No, because you said it was cool.*' _p3_* | C |
|  |  |  | Lafferty 2023^36^ | Offers and resistance | Non-utilisation of services does not necessarily reflect non-offer and people may decline offers of treatment for a multitude of reasons. | The non-judgemental approach of prison health staff facilitated care management among people entering prison. 'Just the way youse come and approached us and talked to us about it and how everything was worked out and that [the treatment] had a 95% success rate and you know hearing that is like good news you know what I mean.' _p1156_ | C |
|  |  |  | Thornton 2018^41^ | Respect | Other prisoners demonstrated respect and trust for peer educators. Peer educators also aspired to adopt a professional demeanour and built rapport with prison staff. | Inside prison we don’t always communicate well with other people, but once you’re an ECHO peer educator you’re more accessible to other inmates. They see you and they know that you have a little more information and they’re not as intimidated to start a conversation as they normally are with the divisions that occur in prison.' 'It made me want to be more professional in the way I acted outside of the work‑ shops away from you guys. It did help me. I built a rapport with a lot of the COs (Correctional Officers) in there.' _p1551_ | U |
|  |  |  | Lafferty 2020^34^ | Linking social capital | Linking social capital (between individuals or groups and institutions) is an important resource for HCV treatment uptake amongst those incarcerated, particularly an ability to trust healthcare personnel within the prison setting. | I freaked out when I found out that I had two different genotypes, like 1a and 3a and I never thought that coud happen you know, so I was a bit worried, but the nurse reassured me and that.' _p5_ '(The SToP-C officer is) good actually. She is good. … She doesn't judge, always has a smile on her face, she tries to help you. She's good. I like her.' _p6_ | U |
|  |  |  | Lafferty 2017^32^ | Linking social capital (connections between inmates and institution) | Linking social capital was a valuable resource for inmates in making decisions about accessing HCV treatment and care. Participants descriptions indicated a degree of trust of clinic staff which in turn enabled participants to express agency and make informed decisions about treatment. | It took [the nurse] a little bit to convince me to do it. And then I  finally done it. And I didn't have no dramas. [But they talked you into it?] They didn't talk me into it, they just explained a few things to me. And you know, give me… didn't push me or anything, just gave me time to think about it and that.' _p3_ | U |
|  |  |  | Jacob 2000^48^ | The relationship between health care staff and prisoners | Prisoners are more likely to confide in outsiders than  in prison staff members who have a control function within the institution. | I also realized that the contact room is particularly important because usually there is no place in prison where drug-using people can speak openly about their problems; we as external service providers [have an advantage]; they have more confidence in us than in internal staff members.' _p334_ | C |
|  |  |  | Lafferty 2022^35^ | Reach | Aspects of patient engagement with the treatment. In this study, the reliance on peer and officer champions. | I find a full-time role in one centre, because you’re all over it then, you know who’s on treatment, who’s doing what, who you’ve seen, oh, there’s new people up in that unit, I better target them, that sort of stuff, so I really find a regular face in the centre, everyone knows you, then all the inmates know you, “Oh, you’re the SToP-C nurse. Give me some jellybeans, ha ha,” and then you go, “Hey, you know, you can get more [discussion] out of them,” and they’ll go, “Oh yeah, my friend and I are sharing [injecting equipment], blah, blah,” and I’ll go, “Are they on the study?” “No,” “Can you just tell them to put their name down?” That’s all I do, but that’s a way of me going, “Shit, I’ll see if he’s got hep C too,” or whatever, but I don’t, you know, I don’t even ask names, I just go, “Is he on the study, your friend?” “Oh no” “Well let’s, you know, get him to put a form in,” just stuff like that, so I think a regular nurse and a regular like Corrections [officer] really would help someone in the role.' _p4_ | U |
|  |  |  | Dyer 2009^27^ | The relationship between health services and prison services | The success of education programs provided depended on the educator's contacts within the prison. | We have a great relationship with the medical staff and the system relies on this communication and rapport. If personnel were to change, there are no formal procedures in place.' _p39_ | C |
|  | 3. Enacted stigma (discrimination against stigmatized people) | Discrimination due to association with drug use | Ly 2018^45^ | Stigma associated with infection and testing | Study participants raised concerns about stereotyping, prejudice and discrimination related to disclosing their status and testing. | My friends and family look down on this. If I get Hepatitis C, then I need to clean it up first. I can't tell my family right away. It is not a good thing to tell them.' _p216_ 'You have to be careful around people, they may not treat you the same.' _p216_ | C |
|  |  |  | Jack 2020^29^ | Stigma | The participants had intense feelings of discontentedness and stigma towards HCV infection. The origins of the stigma were rooted in it's association with injecting drug use. | Yeah, it's difficult because you have to stay away from them like … people avoid them, they do get treated different, people that know they've got hep C like, when smoking was involved, smoking in jail, people won't share fags with them, or share a drink.' _p3_  'It's like a lot of people with hep C are (drug) users and using (drug taking) is looked down on, frowned upon, so that's probably I think, bit of a stigma attached to it, do you know what I mean?' _p3_ | U |
|  |  |  | Lafferty 2018^33^ | Stigma | Within the prison environment, HCV is linked with IDU, leading to stigmatisation, 'being labelled as lesser than or not quite human.' | 'Having hep C is a bad stigma … straight away people link it to intravenous drug use … The stigma of hepatitis, I just want to get rid of it and be done with it … When [other inmates] find out you've got hepatitis … they don't want to be out with you [share a cell] ... [You're] like a pariah.' _p133_ | U |
|  |  |  | Mina 2016^47^ | Stigma | Stigma as a result of being identified as HCV positive and hence as a likely injecting drug user or humiliation of being shackled and in a prison uniform in public waiting areas. | If they come forward, their confidentiality is compromised and this will identify them as an injecting drug user to custodial staff. This will then lead to more searches and more random drug testing - why would they bother?' _p8_ | U |
|  |  |  | Crowley 2018^26^ | Concerns regarding confidentiality | Lack of confidentiality was a barrier to HCV screening and treatment. Often breaches were inadvertent and were related to prisoners being called to attend certain clinics. | It's difficult at times - when prisoners are called to see the Hep (in-reach hepatology) nurse or the phy (methadone) doctor … why else would they be seeing them'. _p4_ | U |
|  |  |  | Jack 2017^28^ | Stigma | The stigma of HCV was due to it's association with intravenous drug use and HCV was a taboo subject amongst the prisoners. Also visits to hospital out-patient clinics were a source of stigma. | there's one on our wing, he was quite embarrassed by it [HCV]  so he told everyone he had cancer.' _p7_ 'and it's always that stigma when you walk in [to a hospital clinic], people look, oh my God it's a prisoner.' _p7_ | U |
|  |  | Discrimination from lack of knowledge | Crowley 2018^24^ | Fear of being stigmatised | Fear of being stigmatised by other prisoners and staff if they became aware of their HCV status. Also stigma of being identified as a prisoner when in a hospital setting. | Then one of them called me aside because it's gone around that I had the virus. That's why I got sacked from the kitchen because I had the virus… Pure ignorance to take me out of the kitchen, just because I had Hepatitis C and at that stage it was gone'. _p4_ | U |
|  |  |  | Lafferty 2023^36^ | Adjudication | The judgements and decisions of health professionals that allow or inhibit continued progression through the HCV cascade. | Concerns of adjudication were apparent within community health settings; a sense of vulnerability of being perceived as a 'junkie'. A participant compared the stigma he felt at a community clinic compared to the acceptance and non-judgemental care he receives from a prison nurse: 'it's part of your [prison nurse] job to deal with it [hep C] you know what I mean? Part of your job not to criticise us for having hep C or stuff like that, like if I'm out [in the community] and I go to a clinic, I feel like they are criticising me whether or not they are, but like either way, that's just the way I feel so I won't even bother.' _p1156_ | U |
|  |  | Privacy and confidentiality | Crowley 2018^24^ | Concerns regarding confidentiality | Concern expressed whether non-medical staff had access to medical records. The process of being called for appointments was not confidential from other prisoners and security staff. | Getting called for tests as you walk onto the landing and there,  you're getting called for blood test and people see you going and say why's he getting a blood test, why why why you know?' _p4_ | U |
|  |  |  | Jack 2020^29^ | Privacy | The prisoner's struggles to deal with a lack of privacy when being screened. The location where testing was carried out was important to many people on prison. | I think the main healthcare. Probably at the same time when people are doing other tests, you know. So it can be purely for the reason that it can be explained, you know, or it can be easily shoved off.' _p3_ 'If people know that hepatitis testing is done on a Wednesday and someone says "oh what are you doing here", but on a Wednesday, it's straight away hep, so the fact that it's a specific nurse and a specific test on that day.' _p3_ | U |
|  |  |  | Munoz-Plaza 2005^39^ | Confidentiality | Talking confidentially to a staff person will "raise eyebrows". | If you are sitting down with staff, [other prisoners] think you are telling [snitching].' _p355_ | U |
|  |  |  | Jacob 2000^48^ | Confidentiality | Many drug users were reluctant to formally declare their participation due to fear over losing anonymity. | ...but there will be no such thing as total anonymity. But that’s got  nothing to do with the implementation of the project; that’s got to do with transparency within prisons, that many things are known.' _p329_ | U |
|  |  |  | Khaw 2007^31^ | Concerns about confidentiality and stigma | There were concerns that confidentiality would not be maintained after accessing healthcare. | I the way, I would think of it is more private. More privacy around it. Because there's a lot of people don't want to know they've got it because when you got to put your sheets in and all that, you come down here with twenty, thirty other people and they're all what ye here for and all the rest of it.' _p3_ 'There was a lass with Hep C on landing 2 and it was "Heppie" and that they call her, do you know what I mean? Yeah. Not very nice.' _p3_ | U |
|  | 4. Duty of care caused conflict | Conflict between confidentiality and staff safety | Crowley 2019^25^ | Conflict between maintaining confidentiality and concerns for personal safety | There was conflict between wishing to maintain  confidentiality for the prisoners and the need to ensure personal safety. This was expressed regarding the presence of prison officers during the committal interview, ward rounds and when taking blood. | There are security issues though; I would not like to do it  without an officer … I feel conflicted.' _p4_ | U |
|  |  |  | Jack 2017^28^ | Confidentiality | The rights of prisoners to expect confidentiality about health issues including their HCV status was widely acknowledged but could create a dilemma for prison officers in deciding whether or when to share the information. | confidential, nothing to do with anybody else apart from  healthcare and the individual.' _p7_ 'There's a lad, got a set of hair clippers and he'll lend it to two or three others on the wing while he had hepatitis, and you just feel like going up to them and giving them a nudge and saying 'listen you really shouldn't be using those shears', but we can't.' _p8_ | U |
|  |  |  |  |  |  |  |  |
|  |  |  |  |  |  |  |  |
| **4.The organisational structure of the prison  and healthcare services** | 1. Stability of prison life. |  | Crowley 2019^25^ | Stability of prison life | Stability of prison life and access to medical care was a facilitator to prisoner's engagement in HCV care. | So the stability … the chaotic lives stop and the support network … can wrap around that individual.' _p6_ | U |
| U = 23 C = 11 14 studies  (8 scored 3 or less out of 5 on dependability) |  |  | Dyer 2009^27^ | Benefit of completing treatment in  custody | It was frequently recognised that completing treatment in custody had advantages. | Undergoing treatment is easier in custodial settings than in the community from a risk perspective … the structured environment makes adherence easier.' _p40_ | C |
|  |  |  | Wurcel 2021^42^ | Impact of captivity and transience on HCV testing | Several felt that jail was a good time to offer testing.  For some, this was the only access to healthcare they had. Transience could be a barrier. | If you come here, get tests, the test is confirmed … if you detox and re-evaluate life … it's a good time in a sense that we have a captive audience.' _p6_ 'The only access to healthcare that a lot of our indigent populations and substance abusers who are at the highest risk of being infected with hepatitis C ... oftentimes is only when they're in jail so feel [testing for HCV in jail] is a necessity.' _p6_ 'This is a transient population, it can be hard to follow treatment from start to finish since it takes 8-12 weeks.' _p6_ | U |
|  | 2. Security requirements impact healthcare access. |  | Byrne 2023^46^ | Prioritisation of work | Security took priority over health care. | Individual custody trumped healthcare in the prison,  hindering improvements to care. _P7_ Clinical staff found it difficult to implement healthcare initiatives as it was perceived as secondary to the regimental running of the prison/security. _p8_ | C |
|  |  |  | Crowley 2019^25^ | Security and safety requirements impacting access to prisoners | Security and safety within prisons placed limitations  on health care delivery. | Security comes first and we may never get to see the  prisoners.' _p3_ | U |
|  |  |  | Jack 2017^28^ | Safeguarding | The need to safeguard the prison's security regime, coupled with ensuring the safety of prisoners and staff was dominant with the health and wellbeing of prisoners considered secondary except in cases of emergency. | You can't treat'em till we get security right. You just cannot.' _p6_ | U |
|  |  |  | Crowley 2018^26^ | Priority of safety and security | While supportive and understanding of the benefits of prison health, the primary focus of prison officers was to ensure the safety of staff and visitors. | Supporting healthcare is important but we need to ensure that staff are covering the landings first.' _p3_ | U |
|  |  |  | Lafferty 2022^35^ | Compatibility | The compatibility of the treatment aim within the prison setting. | Sentence duration can have implications on the implementation of HCV treatment-as-prevention. 'The problem you have with the female offenders is that they don't get the long sentences like the men, so they may be here and they might start something, but then they get released.' _p3_ Security classification also impacted upon movement within the prisons. 'When they’re paged and they’re not coming, makes it difficult in this environment because it’s so free-range, we can’t just go and get somebody, do you know what I mean, whereas in other jails when they’re high security, they’re a bit more contained and you can actually go and get somebody, but in the free-range, we can page people 10 times and they don’t come. _p3_ | U |
|  |  |  | Lafferty 2022^35^ | Perceived effectiveness | The extent to which the intervention is perceived as likely to achieve its purpose. | Due to movement of people in prison, a successful treatment as  prevention intervention was perceived as requiring ‘buy-in’ from the entire prison system. 'I think one of the biggest issues is how much people move about in this system. You know the prisoners they get moved about. I think that’s one of our biggest barriers, that maybe if people stayed in one place for a length of time and we could treat that group of people, you know, there wasn’t as much moving about and different people coming in and out of that group all the time, yeah I think it would work, but I’m not sure because of all this moving about.' _p4_ | U |
|  | 3. The availability and organisation of prison healthcare | Care pathways | Lafferty 2023^36^ | Service permeability | The ease with which patients can access a service, including explicit and implicit gatekeeping. (Includes attitudes of correctional officers). | People in prison, particularly those with higher security classification, rely on officers for movement within prison. _P1156_ '*Do you think officers think testing is a good thing for us to have?* Yes, because they would be worried about getting like pricked or something you know what I mean?' _p1156_ | C |
|  |  |  | Neuhaus 2018^44^ | Adaptability | Adaptability of the care model for the newer treatment. | 'Basically we’ve been left to treat them, once they’ve been started on treatment we have been left as nurse practitioners, which is good, to treat them on an everyday basis. And we don’t need to kind of check in with the gastro about them anymore unless we feel that we need to. ' _p694_ | C |
|  |  |  | Crowley 2019^25^ | Inter-prison variations in prisoner health needs and health service delivery and priority | The heterogeneity of prison populations was linked with differences in how health care was delivered at different locations. | It is very urban centric and then you get out of the urban areas … more rural … and we are not as invested in and it is not as high profile.' _p5_ | U |
|  |  |  | Crowley 2018^26^ | In-reach hepatology and fibroscanning services | The availability of in-reach hepatology and mobile elastography were seen as enablers to prison engagement in HCV care. | Anything that stops us going outside the main gate is good'. _P6_ 'Certainly, the awareness of the Hep C has gone up in the last couple of months with the fibroscanner … I see more engagement with staff and prisoners.' _p6_ | U |
|  |  | Availability of healthcare | Crowley 2019^25^ | Positive impact of in-reach hepatology services | In-reach hepatology services had a positive impact on linkage to care and the added benefit of increasing awareness of HCV. | In reach has transformed that. It's hugely important, that accessibility. That changes how people think. It's foremost in peoples minds. They're being seen. That makes a huge difference.' _p5_ | U |
|  |  |  | Neuhaus 2018^44^ | Organisational impacts | These include cost, staff knowledge and confidence, staff time and workload. | Well the way with the hepatitis C prescriptions is that there’s quite a delay in getting it in from central pharmacy. So we have to rearrange our schedule and make sure that we place the order well before Monday in order to get the delivery supply the Friday of the same week. ... We try to work in a schedule so that we get the prescriptions and check through them for validity and ordering them before the deadline.' _p694_ | U |
|  |  |  | Neuhaus 2018^44^ | Access to therapy | Due to limited access to specialists, most prisoners had poor access to HCV treatment prior to the telementoring service. | So we never, we weren’t treating anyone, we had not treated a single prisoner, as far as I’m aware, that wasn’t acute kind of thing, in our prisons prior to us doing this. Yet we’d had medication for 12 months available to us.' _p694_ | U |
|  |  |  | Jacob 2000^48^ | Acceptance of the intervention | Availability and acceptance of harm reduction measures eg sterile syringes and education. | The project can be considered a success, because no negative side  effects occurred, except for some minor technical failures and particularly because it pretty fast became something natural, a part of everyday life; it was nothing special anymore.' _p329_ | C |
|  |  | Healthcare constrained by prison organisation | Byrne 2023^46^ | Organisational culture | The work of clinical staff was constrained  by the nature of how they could operate within the prison. | Clinical staff found it difficult to transit individuals to  the prison health centre due to the provision of opioid addiction treatment at concurrent times._p7_ A lack of freedom for health care staff to operate in the prison hindered the design of the pathway. _p7_ The lack of physical space and clinic rooms adversely affected how and when the test could be used. _p7_ As nurses were not present for the majority of admissions, in-reach was limited_. p7_ | U |
|  |  |  | Crowley 2019^25^ | Linkage to care due to length of prison  sentence | Challenges were reported with linkage to care due to short prison sentences served. | They're hardly in but they are gone home … what do we do with the positive test then.' _p5_ | U |
|  |  | Healthcare leadership ethos | Byrne 2023^46^ | Leadership | Leadership could hinder or facilitate the care pathway. | Clinic staff found it difficult to engage colleagues outside their direct team due to a perceived lack of integrated care. _P7_ Clinic staff found it easier to engage patients due to the ethos of their team which values individual relationships. _p8_ | C |
|  |  |  | Byrne 2023^46^ | Self-efficacy | An openness to change and a perception  that the test would ease workloads were facilitators. | You've got the difference between getting a result you  can act upon, rather than having to wait a week. So, that's a major advantage.' _p9_ | C |
|  | 4. The timing of the intervention |  | Crowley 2018^24^ | Opt-out screening at committal | Screening on committal was seen as more private, but others concerned this would add stress at an already stressful time. | Make it automatic when you come in on committal'. _P5_ 'A big group to do everyone there and then people are coming in with withdrawals… it's a difficult situation for us to be in too.' _p5_ | U |
|  |  |  | Khaw 2007^31^ | Lack of proactive approaches to offering testing | The offer of testing may not arise at an appropriate time. | Prisoners with short sentences might not be able to keep their appointments for testing because of court appearances or early discharge. _P4_ 'I was just trying to get through my rattle [detox]. That's all I were thinking about.' _p4_ | C |
|  |  |  | Crowley 2019^25^ | Timing of screening | There were mixed views on timing of screening. Some favoured opt-out at committal, others expressed concern about prisoners having competing priorities at this time. | One of the things to get rid of the barriers is making it routine at committal, you wouldn't think twice. That's probably where we need to get to. The same way as you expect to give urine.' _p5_ 'They're all actively seeking medication … screening blood tests is so far down the line of what they are thinking. Day after still all over the place ... two weeks later better when they're settled...' _p5_ | U |
|  |  |  | Crowley 2018^26^ | Time of screening | Participants favoured a structured and systematic approach to HCV screening with the committal period identified as an opportune time. | Committal is a good time to get them tested … it's always staffed, in fact you should make it compulsory … of course, unless they know their test result already'. _p5_ | U |
|  | 5. Limited custodial and healthcare resources |  | Munoz-Plaza 2005^39^ | Limitations in correctional funding | The prison was pulling back on its support of HCV testing services due to funding concerns. | The majority said the budget is always a problem. _P357_ Inmates said they began to sense that correctional officials became worried that targeted peer education efforts to encourage testing were resulting in an increase in the number of inmates asking to be tested. _p357_ | C |
|  |  |  | Crowley 2019^25^ | Staffing and rostering issues | Lack of staffing was a barrier to the provision of  health care in prisons. Other duties were prioritised over blood borne virus screening during periods of low staffing. | Generally happens at the start of each pool of overtime hours and rarely is the activity done at the end when you're really short and you're barely giving out the medication. So it's quite sporadic and opportunistic.' _p3_ | U |
|  |  |  | Mina 2016^47^ | Lack of capacity in prison health service. | HCV was not considered a health priority by the prison health service, nor were there enough custodial officers available to transfer inmates to hospital based services. | 'Prison health is very resource-limited - any extra burden on staff is not welcome.' _p6_ 'There is high staff turnover and a continual need to train people.' _p7_ 'There was only one specialist nurse who works ten hours per week responsible for initiating treatment across the eight prisons in the state.' _p10_ | U |
|  |  |  | Dyer 2009^27^ | Staff availability | In seven of the eight states and territories mentioned, the position responsible for a hepatitis related service had been vacant and thus the program had lapsed. | The most important thing is for programs to be sustainable .. Keeping staff is difficult.' _p39_  'The rotation and turnover of staff makes it challenging to reach everyone.' _p39_  'The prison population is linguistically diverse … getting translators to deliver these services is difficult.' _p40_ | U |
|  |  |  | Byrne 2023^46^ | Readiness to implement the intervention | Staff turnover and training issues  impacted the intervention. | Staff found it difficult to plan a '1 day' test/treat  pathway due to safety concerns with the frontline medication used. _P7_ Clinical staff indicated a preference to obtain a venous sample to fingerprick sample due to their self-perceived proficiency at obtaining venous bloods. _p7_ | C |
|  |  |  | Lafferty 2022^35^ | Reach | Aspects of patient engagement with the treatment. In this study, the reliance on peer and officer champions. | Although dedicated officers had a leading role in implementing the  intervention, it was widely viewed that whole-of-officer workforce was important for scale up efforts. | C |
|  |  |  | Crowley 2018^26^ | Staffing and resources | Lack of custodial staff impacted negatively on the delivery of healthcare. | I think it's … it's looking at how you maximise operations, … the most efficient system in place and how to support that. Whether it's more officers or nurses. There should be more supports in the prison and resources should be going into the prison rather than spending it on sending prisoners back and forth to the hospital'. _p3_ | U |
|  |  |  | Lafferty 2022^35^ | Effectiveness and efficacy | Securely funded positions for dedicated personnel and continuity of care for patients transferring between prisons. | Well, the fact that – because the University is paying for this, that’s a major – because we, if we don’t have enough officers, we’ll have posts like stripped or closed down whereas because this is run by the University, so they’re actually paying for our time. […] Even if we’re 20 [officers] short, they can’t redirect me because I’m not under Corrective Services guidelines today, like I’m at the University’s disposal, so that’s been good in ensuring that it does continue to happen because that is a big issue with – we could run as many get-fit programs as you wanted through Activities, but they would fail because Activities is the first to be stripped.' _p3_ | U |
|  |  |  | Lafferty 2022^35^ | Burden | The perceived amount of effort that is required to participate in the intervention. | Most correctional participants (both front-line and management)  viewed the intervention as having negligible impact on their workload, notably because of the availability of a dedicated officer to facilitate patient movements. p3  '[Have there been any disadvantages of SToP-C?] Not from our half […] I don’t think so at all because I mean you have the one officer allocated to it and they just take care of it. That’s their job for the day, so it’s pretty good really.' p4 | U |
